# A cognitive architecture for Long COVID: the interplay of brain fog, fatigue, attention and memory

**DOI:** 10.1093/braincomms/fcag361

**Published:** 2026-09-24

**Authors:** Darragh Higgins, Sijia Zhao, Masud Husain, Peter Denno, Peter J Hellyer, William Trender, Christina J Atchison, Bethan Davies, Emily Cooper, Joshua Elliott, Matthew Whitaker, Chiara Di Gravio, Marc Chadeau-Hyam, Paul Elliott, Helen Ward, Adam Hampshire

**Affiliations:** Department of Brain Sciences, National Institute for Health Research Biomedical Research Centre, Imperial College London, London W12 0NN, UK; Centre for Neuroimaging Sciences, Institute of Psychiatry, Psychology and Neuroscience, King’s College London, London SE5 9RS, UK; Department of Experimental Psychology, University of Oxford, Oxford OX1 3PH, UK; Department of Experimental Psychology, University of Oxford, Oxford OX1 3PH, UK; Centre for Neuroimaging Sciences, Institute of Psychiatry, Psychology and Neuroscience, King’s College London, London SE5 9RS, UK; Department of Brain Sciences, National Institute for Health Research Biomedical Research Centre, Imperial College London, London W12 0NN, UK; Centre for Neuroimaging Sciences, Institute of Psychiatry, Psychology and Neuroscience, King’s College London, London SE5 9RS, UK; Department of Brain Sciences, National Institute for Health Research Biomedical Research Centre, Imperial College London, London W12 0NN, UK; Department of Epidemiology and Biostatistics, School of Public Health, Imperial College London, London W12 0BZ, UK; Department of Epidemiology and Biostatistics, School of Public Health, Imperial College London, London W12 0BZ, UK; Department of Epidemiology and Biostatistics, School of Public Health, Imperial College London, London W12 0BZ, UK; Department of Epidemiology and Biostatistics, School of Public Health, Imperial College London, London W12 0BZ, UK; Department of Epidemiology and Biostatistics, School of Public Health, Imperial College London, London W12 0BZ, UK; Department of Epidemiology and Biostatistics, School of Public Health, Imperial College London, London W12 0BZ, UK; Department of Epidemiology and Biostatistics, School of Public Health, Imperial College London, London W12 0BZ, UK; Department of Epidemiology and Biostatistics, School of Public Health, Imperial College London, London W12 0BZ, UK; UK Dementia Research Institute at Imperial, School of Public Health, Imperial College London, London W12 0BZ, UK; Department of Epidemiology and Biostatistics, School of Public Health, Imperial College London, London W12 0BZ, UK; Centre for Neuroimaging Sciences, Institute of Psychiatry, Psychology and Neuroscience, King’s College London, London SE5 9RS, UK

**Keywords:** cognitive assessment, Long-COVID, subjective symptoms

## Abstract

The cognitive mechanisms underlying post-acute COVID-19 sequelae (‘Long COVID’) remain poorly defined. While deficits in memory and executive function are well-documented, it is unclear whether these arise from primary neural dysfunction or are secondary consequences of fatigue-related factors. Here, we addressed this in a community cohort (*N* = 24 095) using a custom cognitive battery to dissect the interplay between sustained attention, fatigue, and memory. Among this subset, 11 925 participants had previously tested positive for COVID-19, and 548 reported ongoing symptoms consistent with Long COVID. Path analysis (structural equation modelling) was used to evaluate competing mechanistic models linking COVID-19-related features (e.g. duration of symptoms, severity of illness) with objective performance and subjective cognitive symptoms. Participants with persistent symptoms post-COVID-19 (unresolved beyond 12 weeks) showed significant deficits in sustained attention compared to non-infected individuals. Path models revealed complex relationships underlying these deficits. Unlike models of mental health disorders and general ageing, where cognitive performance was largely mediated by relationships to fatigue, the final Long COVID model represented a hybrid mechanism: COVID-19 features were associated with direct, independent effects on attention and memory, while simultaneously relating lower cognitive performance to on-task fatigue build-up. Findings also suggest that ‘brain fog’ reflects a distinct neurocognitive vulnerability associated with fatigue and reduced performance on sustained attention and memory tasks. These findings accord with the hypothesis that Long COVID has a heterogeneous, multi-faceted impact on cognitive function, with higher-order processes such as memory being particularly vulnerable due to their dependence on foundational attention and motivation processes.

## Teaser

Higgins *et al*. report findings from a large dataset of COVID-19 patients. The findings show deficits in memory and attention specific to patients with Long COVID. Structural equation modelling is used to highlight how on-task fatigue and subjective symptom measurements also contribute to post-COVID-19 cognitive impairment.

## Graphical Abstract

**Graphical Abstract:**
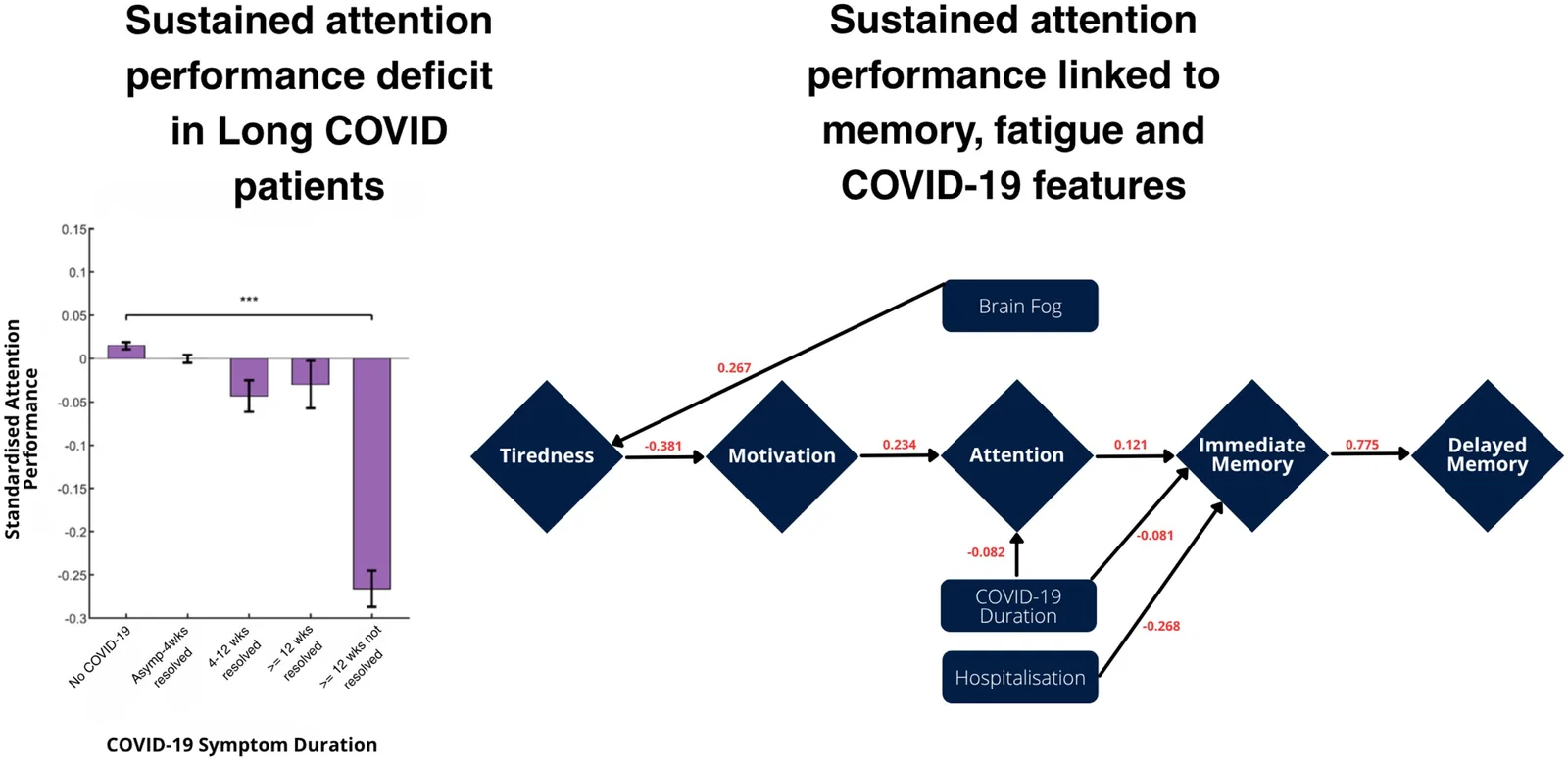

## Introduction

Neurological symptoms associated with COVID-19 were first recognized early in the pandemic.^1,2^ Although numerous studies have attempted to explain the biological mechanisms by which severe acute respiratory syndrome coronavirus 2 (SARS-CoV-2), the virus causing COVID-19, may affect the central nervous system and cause neuropathology,^3-8^ there remains an important gap in our understanding of the condition’s long-term cognitive effects. Although studies have previously reported objectively measurable cognitive deficits following SARS-CoV-2 infection, particularly in attention, memory and reasoning performance,^9-12^ they have not established how these deficits relate to each other and to COVID-19 sequelae such as acute illness severity, chronic brain fog and fatigue, which may affect cognitive performance.

‘Long COVID’ and ‘post-COVID Syndrome’ are terms most commonly attributed to the subset of individuals who report symptoms beyond the acute phase of COVID-19.^13^ This condition is typically defined by persistent symptoms that impact physical and cognitive functioning ≥12 weeks post-infection.^14^ Estimates of Long COVID prevalence vary. Some studies suggest up to 45% of COVID-19 survivors experience at least one symptom 4 months or more post-infection, regardless of hospitalization.^15^ Others report a lower but still substantial prevalence of persistent symptoms: 10–30% in non-hospitalized patients and 50–80% in hospitalized patients.^16^ The nature of symptoms can also vary, with fatigue, pulmonary and vascular problems, anosmia, dysgeusia, myalgia and neurocognitive issues among the most common.^17-19^ Subjective psychological symptoms are also frequent, with studies highlighting ‘brain fog’, depression, anxiety, mood and psychotic disorders, perceived cognitive decline and distractibility.^20-29^ Brain fog in particular has generated significant interest, as it is reported by 20–30% of patients with persistent symptoms, though the term’s clinical specificity remains ambiguous.^24,30^ There is some evidence of milder brain fog symptoms in patients infected during the Omicron-dominant epoch compared to earlier variants,^11,31^ likely reflecting both vaccine protection and viral evolution.^10^

A substantial body of evidence now supports the view that COVID-19, particularly in cases of persistent symptoms, is associated with cognitive performance deficits measured via objective tasks.^10,22,23,26^ SARS-CoV-2 variant epoch, illness severity, illness duration, hospitalization and cognitive symptoms such as brain fog have also been linked to cognitive deficits.^12,20,21^ However, the relationship between attention and impairment in other cognitive domains remains unclear. Deficits in fundamental attentional processes may impact higher cognitive functions such as memory and reasoning if these domains are impaired via hierarchical mechanisms; relatedly, if persistent post-COVID-19 symptoms such as fatigue or brain fog influence attention, they may impact memory processes by extension. Alternatively, the impact of COVID-19 features on different domains of cognitive function could occur in parallel, with attentional, memory and reasoning systems all affected somewhat independently. There may also be population heterogeneity regarding which domains are affected and the manner in which these effects manifest. Moreover, the relationship between objectively measured deficits and subjective cognitive symptoms is complex. Among those with ongoing persistent symptoms, reports of brain fog and memory symptoms correlate with poorer performance on memory, attention and executive function tasks.^32-34^ However, these associations are modest and diminish in patients whose symptoms have resolved.^24^

Post-viral fatigue is another key potential contributor to cognitive symptoms and performance deficits.^32^ Two subjective dimensions of fatigue (tiredness and motivation) are often studied alongside sustained-attention performance.^20,35-37^ Tiredness is assessed as part of the broader fatigue assessment.^10,26,38^ Both attention and tiredness are inversely related to motivation and may be conceptualized in physical or cognitive terms. Physical and cognitive fatigue both associate with reductions in motivation and attention.^39^ Motivation is central to allocating resources needed for cognitive systems,^40^ thereby modulating memory and attention during goal-oriented behaviour.^36,41-43^ It remains unclear though how this interplay among fatigue, symptoms and cognitive deficits operates in Long COVID.^44-46^ Thus, there is a need to move beyond isolated domain-specific impairments by considering interrelations across objectively measured cognitive functions and subjective assessments of tiredness and motivation.

Similar questions have been addressed using structural equation modelling (SEM), which allows comparison of alternative models of variable relationships using methods such as path analysis or confirmatory factor analysis, among others.^47,48^ SEM has been used to link pre-existing health conditions, psychiatric symptoms and COVID-19 severity;^49,50^ post-COVID-19 pain sensitivity, Long COVID and mental health;^51^ and COVID-19 risk factors, cognitive flexibility and sleep quality.^52^ We aimed to advance these applications of SEM by using path analysis to adjudicate between hypothesis-driven models connecting COVID-19 features, symptoms and cognitive deficits.

## Materials and methods

### Participants

We analysed data from the REACT Long Covid study (REACT-LC).^53,54^ REACT-LC extended the REaltime Assessment of Community Transmission (REACT) cohort, which tracked the prevalence of infection with SARS-CoV-2 across England from 1 May 2020 to 31 March 2022. The analysis included data from a randomly selected community sample of 3 099 386 adults (≥18 years of age). Of these, 2 494 309 participants consented to be recontacted and to allow data linkage with the National Health Service (NHS).

Details of the recruitment process for REACT and REACT-LC are available in^53^ (see Table 1 for demographic information). In brief, 800 000 REACT cohort members were contacted between 1 August and 30 December 2022 to undertake an online study including a questionnaire collecting information about illness features, acute and chronic symptoms, demographics, clinical outcomes, and objectively measured cognitive performance. On completion of the questionnaire, participants were invited to undertake an online cognitive assessment battery on the Cognitron platform. Among the 276 840 respondents who completed the questionnaire, 141 583 started the battery (completed at least one task), and 112 964 (79.8%) completed all eight main tasks. From this latter subset, 31 760 participants were presented with the Digit Vigilance Task (DVT) at the end of the assessment battery, with 24 095 successfully completing it. This task was designed to examine the roles of sustained attention, tiredness and motivation in cognitive performance in more detail. The mean age of participants included in these analyses was 54.73 years, comprising 14 605 females and 9490 males. We previously published analyses of post-COVID-19 performance deficits for the eight main tasks.^10^ Here, we undertake more detailed analyses including the additional DVT. We also analyse results from Immediate and Delayed Object Recognition Memory, as these were the most sensitive tasks in the previous analysis. For all participants, tasks were presented in a fixed order (Immediate Memory first, Digit Vigilance second, Delayed Memory third, within the battery of eight tasks) and completed on personal devices (desktop computer, laptop computer, tablet or smartphone).

**Table 1:** Demographics and group distribution for those who completed the original task battery alongside those who completed both attention and memory tasks

	Completed attention and memory tasks (*N* = 24 095)		Completed original task battery (*N* = 112 964)		Began original task battery (*N* = 141 583)	
	Count	Per cent (%)	Count	Per cent (%)	Count	Per cent (%)
**Age**						
18–24	589	2.44	2541	2.25	3607	2.55
25–34	1442	5.98	6844	6.06	9332	6.59
35–44	2752	11.42	12 956	11.42	16 695	11.79
45–54	4561	18.93	20 852	18.46	25 764	18.19
55–64	6866	28.49	31 648	28.02	38 614	27.27
65–74	6032	25.04	28 274	25.03	34 773	24.56
75+	1853	7.69	9849	8.72	12 798	9.05
**Sex**						
Female	14 605	60.61	68 230	60.40	85 193	60.17
Male	9490	39.39	44 729	39.60	56 384	39.82
Unknown	0	0	5	0.00	6	0.00
**Education**						
A Levels	3399	14.11	10 570	9.36	13 620	9.62
Degree	12 124	50.32	37 963	33.61	46 632	32.94
General Certificate of Secondary Education <5	821	3.41	2517	2.23	3311	2.34
General Certificate of Secondary Education >5	2819	11.70	8827	7.81	11 433	8.08
Higher Education	2631	10.92	8441	7.47	10 741	7.59
None	635	2.64	2107	1.87	2883	2.04
Other	1232	5.11	4102	3.63	5276	3.73
Unknown	434	1.80	38 437	34.03	47 687	33.68
**Ethnicity**						
Asian	493	2.05	2351	2.05	3205	2.26
Black	137	0.57	635	0.57	884	0.62
Mixed	276	1.15	1277	1.15	1611	1.14
Other	143	0.59	675	0.59	888	0.63
Unknown	213	0.88	1010	0.88	1316	0.93
White	22 833	94.76	107 016	94.73	133 679	94.42
**Deprivation Quintile (IMD_Q)**						
1 (most deprived)	1866	7.74	8480	7.51	10 912	7.71
2	3482	14.45	16 034	14.19	20 380	14.39
3	5102	21.17	23 564	20.86	29 635	20.93
4	6089	25.27	28 273	25.03	35 267	24.91
5 (least deprived)	7538	31.28	42 136	30.13	42 136	29.76
Unknown	18	0.07	2582	2.29	3253	2.30

A Public Advisory Group has been actively involved throughout the conduct of the REACT-LC study. Ethics approval for the study was received from the South Central–Berkshire B research ethics committee of the NHS Health Research Authority. Participant consent was obtained according to the Declaration of Helsinki.

### Cognitive tasks

#### Sustained attention task

To assess sustained attention and task-induced fatigue, we employed the DVT, an online adaptation of the vigilance paradigm.^55,56^ This specific iteration of the task (also referred to as the Number Vigilance Test) has previously demonstrated high sensitivity to cognitive deficits in the post-acute and chronic phases of COVID-19.^19,20^

The task required participants to monitor a rapid stream of noise-masked digits (0–9) and respond via mouse/screen click only when the target ‘0’ appeared (with 25% probability).

Different from the previous online version used in refs 19 and 20 to standardize difficulty across individuals, the main task was preceded by a 90-trial calibration block utilizing an adaptive staircase procedure. This determined the specific mask opacity threshold required for each participant to achieve an initial target detection accuracy of ∼80%. The calibration block was excluded from the analysis.

Following calibration, participants completed four task blocks (60 trials per block). In each trial, a digit was presented centrally for 50 ms with a stimulus onset asynchrony of 1000 ms. To track the trajectory of subjective symptoms relative to objective performance, participants rated their current tiredness and motivation on visual analogue scales at baseline and during the 40-s inter-block intervals (Fig. 1). This measurement treated tiredness and motivation as twin elements of state-dependent fatigue.

**Figure 1:**
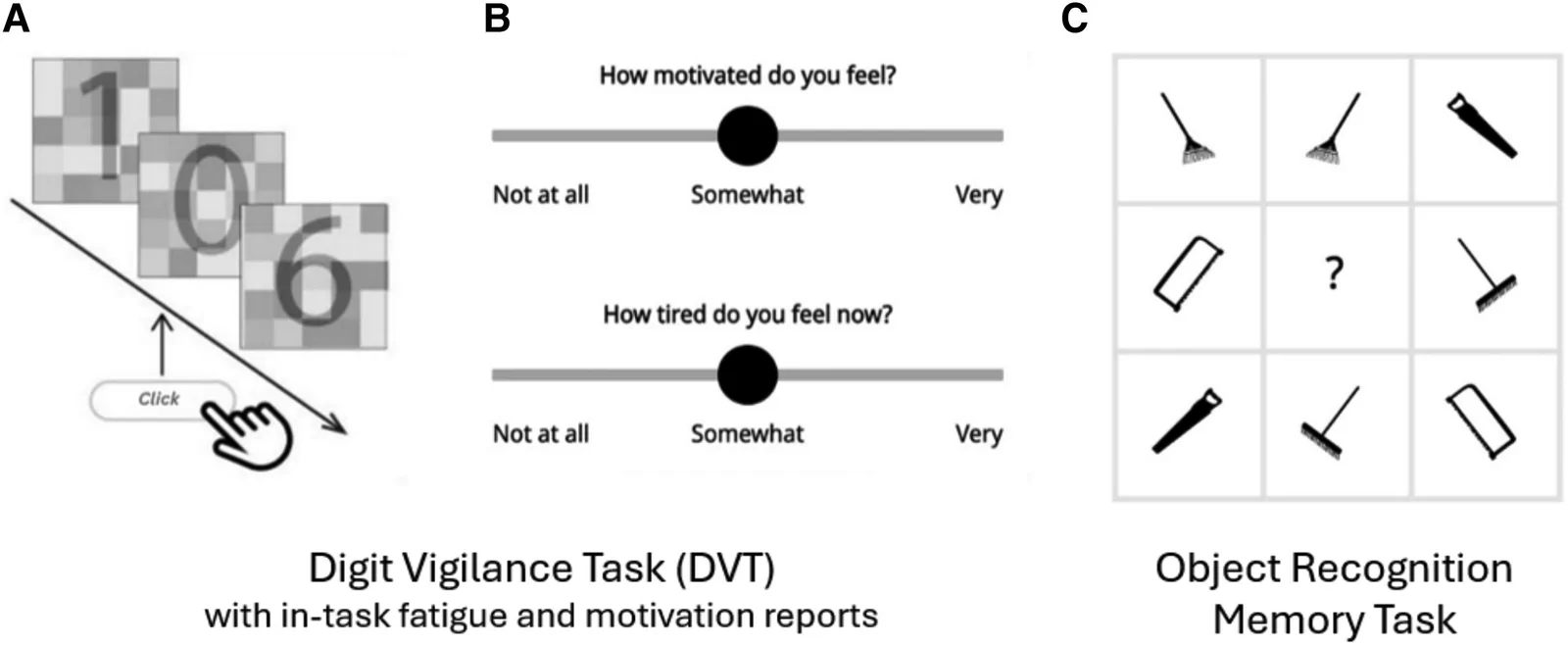
**Cognitive task paradigms.** (A) Schematic of the Digit Vigilance Task (DVT). Participants monitored a rapid serial visual presentation of noise-masked digits (0–9) and were instructed to respond only to the target ‘0’. **(B)** In-task subjective ratings during DVT. Visual analogue scales were presented at baseline and during inter-block intervals to assess instantaneous tiredness and motivation. **(C)** Object Recognition Memory Task. Participants viewed a matrix containing the target object, distractors (category or item errors), and a binding lure (correct object, incorrect orientation) and selected the item that matched the originally encoded stimulus. Figure adapted from Zhao *et al*. (2022) with permission.

#### Memory tasks

Analyses of the immediate and delayed object recognition memory tasks have been reported elsewhere.^10^ For the immediate memory test, 20 images were displayed with an encoding time of 2000 ms and inter-stimulus interval of 500 ms. After the full sequence was finished, each stimulus was then presented within a grid of object silhouette pictures, including the target and seven distractors. The distractors were categorized by how similar they were to the target. Specifically (i) the same picture as the target but with the orientation flipped left-right (binding error), (ii) a picture of an object of the same type—e.g. if the target were a chair, then a different chair (category error), (iii) a different type of object (item error). The main outcome measure was the number of points accrued, where points are awarded for each trial for item error = 0, category error = 1, binding error = 2, correct target = 3, summed across trials. Secondary scores include counts of incidences of each error type and median response times.

After the participants underwent the immediate memory task described above, they completed the other tasks in the battery, which measured vigilance, visuospatial processing, working memory, spatial planning, verbal reasoning, decision impulsivity and knowledge of rare word definitions^10^ before they were presented with object recognition memory test grids as per the immediate memory task, without the encoding period. They were asked to indicate the stimuli they had been asked to memorize at the beginning of the test battery. The main score was the total number of points accrued as described above for immediate memory.

### History of SARS-CoV-2 infection and COVID-19

Details of the definitions and classification of COVID-19 features are reported in.^10,57^ In brief, participants were categorized into six categories based on the presence, duration, and persistence of SARS-CoV-2-related symptoms. Category 1 included participants who self-reported either never having COVID or had an unconfirmed COVID-19 diagnosis. Categories 2–6 required positive results on both PCR and lateral flow immunoassays (68) with symptom duration determined by self-reports. Hence, COVID-19 categories were organized as follows: Category 1 comprised participants with no confirmed SARS-CoV-2 infection; Category 2, asymptomatic infection; Category 3, resolved symptoms lasting <4 weeks; Category 4, resolved symptoms lasting 4–12 weeks; Category 5, resolved symptoms lasting ≥12 weeks; and Category 6, ongoing symptoms persisting ≥12 weeks at the time of cognitive assessment. Infection dates were estimated from symptom onset or, for asymptomatic cases, the date of a positive test. Predominant variant periods were assigned according to the dominant UK strain at infection (wild-type, Alpha, Delta, or Omicron). Vaccination status was defined as receipt of any SARS-CoV-2 vaccine ≥14 days before infection. Hospitalization severity was classified by NHS data linkage as emergency department attendance, hospital admission, or intensive care unit (ICU) admission. The sample size for each of these categories is shown in Table 2.

**Table 2:** COVID-19 features for participants who completed the original task battery alongside those who completed both attention and memory tasks

	Completed attention and memory tasks (*N* = 24 095)		Completed original task battery (*N* = 112 964)		Began original task battery (*N* = 141 583)	
	Count	Per cent (%)	Count	Per cent (%)	Count	Per cent (%)
**COVID-19 duration**						
No COVID-19	12 170	50.51	46 261	40.95	58 173	41.09
4–12 weeks resolved	688	2.86	3246	2.87	4065	2.87
≥12 weeks not resolved	548	2.27	2580	2.28	3103	2.19
≥12 weeks resolved	303	1.26	1350	1.20	1692	1.20
Asymp–4 weeks resolved	10 386	43.10	48 826	43.22	61 280	43.28
Unknown	0	0	10 701	9.47	13 270	9.37
**COVID-19 variant**						
Omicron	6442	26.74	43 323	64.95	53 790	64.49
Alpha	1054	4.37	4812	7.21	6102	7.32
Delta	2933	12.17	14 942	17.91	14 942	17.91
Wild	1426	5.92	6251	9.37	7912	9.49
Unknown	70	0.29	479	0.72	664	0.80
**Hospitalization**						
Hospitalized	162	0.67	527	0.47	649	0.61
ICU	44	0.18	228	0.20	282	0.20
A&E	155	0.64	672	0.59	868	0.61
**Vaccination (doses)**						
0	14 757	61.25	77 013	68.17	96 425	68.10
1	219	0.91	731	0.65	989	0.70
2	9119	37.85	35 220	31.18	44 169	31.20

### Statistical analysis

Data cleaning and adjustment for nuisance variables included the following steps. After removing those who did not fully complete both the sustained attention and object memory tasks within this battery, the dataset contained 31 760 participants. Those who returned an unknown COVID-19 duration (i.e. as were still symptomatic and within the first 12 weeks of infection) or had missing data from reaction time measurements during the attention task were removed, leaving 24 095 participants in the analysis. Linear regression adjusted attention and memory accuracy scores to account for associations with age, gender, ethnicity, level of education, COVID-19 vaccination status and Index of Multiple Deprivation score.^58^ Residuals from this model were standardized using an inverse rank transformation to a normal distribution. Comorbidities were included as part of a sensitivity analysis (see Supplementary Tables 1–3 in Section 1 of Supplemental Data) and did not reveal any material changes to regression results. Distribution figures are included as Supplementary Figs 1 and 2 in Section 2 of the Supplemental Data.

We first confirmed associations of COVID-19 features with object memory performance as reported in our previous analyses of the larger cohort.^10^ Then analyses were extended to the DVT. Specifically, we undertook mixed effects analysis of variance with block as the within-subject factor to determine how attention performance (task accuracy and reaction time), tiredness and motivation changed with time on task. COVID-19 features were included as between-subject factors with two-way interactions to measure their relationship to changes in performance across blocks.

Four separate linear regression models were deployed, with outcome variables of (a) reaction time on the DVT task, (b) DVT accuracy, (c) immediate memory accuracy, and (d) delayed memory accuracy. In all models, predictors included mean ratings of tiredness and motivation, which were averaged across the entire vigilance task, and COVID-19 features (duration, variant period, and hospitalization status). General trait-like fatigue was not included as a measurement in these analyses. For the attention models, DVT reaction time and DVT accuracy were also included as predictors of each other. For the memory models, DVT accuracy was additionally included as a predictor to test whether attention associates with memory performance. Multiple comparisons were used to evaluate point estimates and confidence intervals, with the Bonferroni correction applied to control the family-wise error rate when reporting *P-*values.

We undertook three stepwise regression models to analyse each memory and attention task after inclusion of subjective symptoms reports, to further explore if any two-way interactions had significant associations with task scores. As well as the above predictors, subjective symptom reports of brain fog, memory problems and fatigue were included in these models. The stepwise addition and removal of terms from the linear model was calculated according to Bayesian information criteria (BIC), adding a term if BIC < 0 or removing it if BIC > 0.01.

Finally, path analysis, a subtype of SEM, was performed to evaluate evidence in favour of models representing alternative mechanistic hypotheses of how COVID-19 features may modulate cognitive task performance, via direct paths and symptom features. The models included factors of subjective symptoms (brain fog and memory problems), subjective cognitive measures (tiredness and motivation reports), COVID-19 symptom duration, hospitalization status, and cognitive performance on attention and memory tasks.

Specifically, we constructed four models (see Fig. 2) that were designed to analyse different potential paths between COVID-19 features, subjective symptom data and objective cognitive data, with these models being structural variants of two main hypotheses:

**Figure 2:**
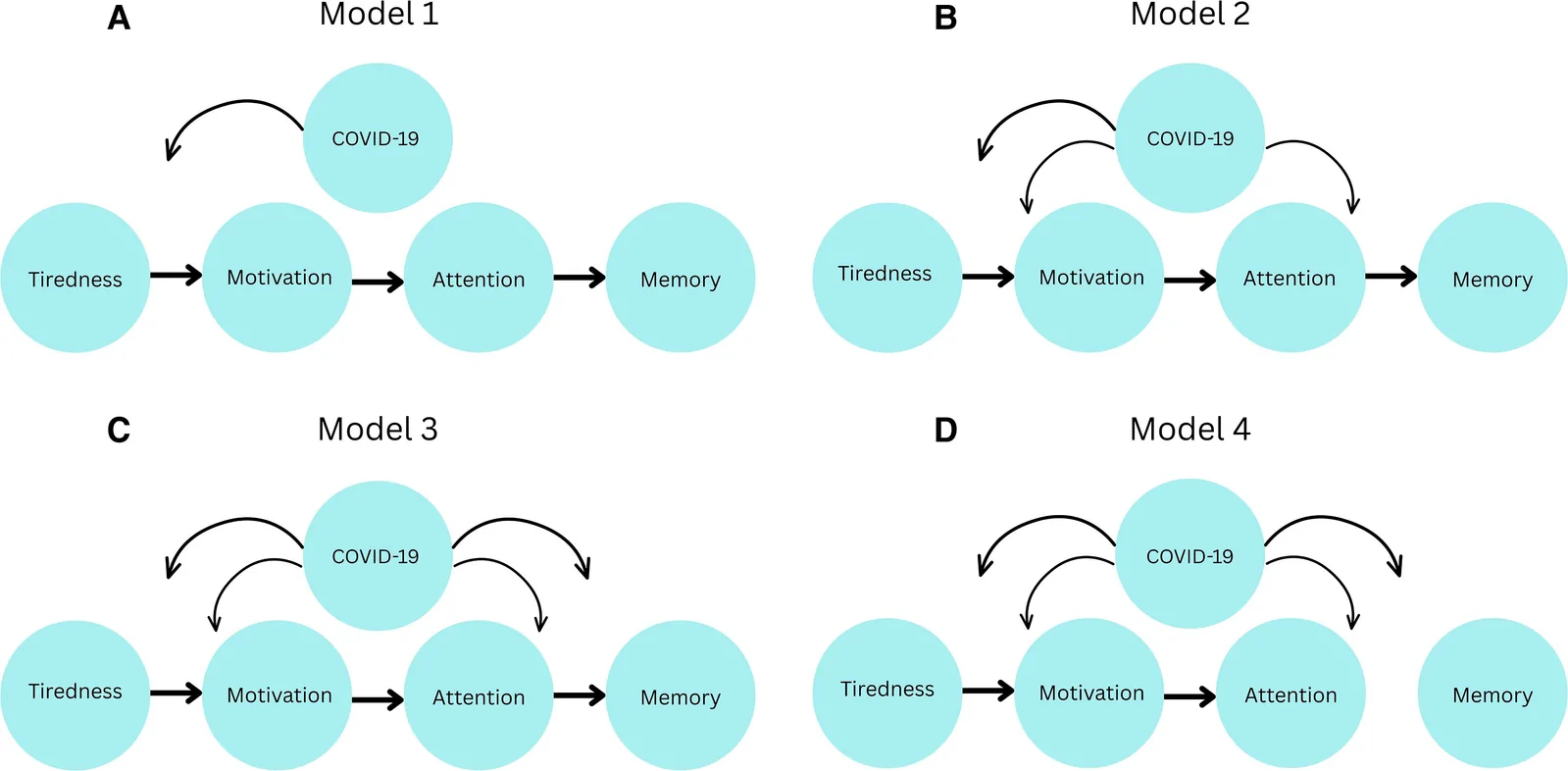
**Structured path models.** Four path models constructed to assess the relationship between COVID-19 features and cognitive performance. COVID-19 features (top circles) were composed of symptom duration, subjective symptom reports and hospitalization status. **(A)** HPM 1: Direct mechanistic path from COVID-19 features to DVT task tiredness, with chain of indirect effects leading through other cognitive domains. **(B)** HPM 2: Partial mediation; chain of effects including direct as well as indirect COVID-19 influences and mediation for tiredness, motivation and attention. **(C)** PPMl 1: Fully direct mediation between COVID-19 features with a chain from tiredness to motivation, attention and memory. Includes both a chain of mechanistic relationships between dependent variables and a stepwise influence of COVID-19. **(D)** PPM 2: Retains the direct mediation of COVID-19 features, but instead breaks the chain of cognitive domains between attention and memory.

**H1—**The Hierarchical Paths Model (HPM), posits that cognitive deficits associated with Long COVID follow a hierarchical pathway, in which fatigue plays a central role. Long COVID leads to increased fatigue, which in turn reduces attention, while reduced attention then impairs memory performance. The effects of Long COVID on memory are thus explained indirectly, via fatigue and attention.

**H2**—The Parallel Paths Model (PPM) posits that associations between Long COVID and cognitive outcomes—including attention deficits, memory deficits, fatigue and subjective cognitive symptoms—are largely independent of one another, reflecting parallel pathways.

**Model 1**, the first Hierarchical Paths Model, was the simplest model. It proposed a direct mechanistic path from COVID-19 through each dependent variable, with the paths modelled as influenced directly by COVID-19 duration, hospitalization and symptoms as mediated by tiredness.

**Model 2** represented partial mediation, proposing a chain of direct and indirect paths from COVID-19 features to cognitive performance, with its influence on memory task accuracy mediated by tiredness, motivation and attention. It was the second Hierarchical Paths Model.

**Model 3** was constructed to assess fully direct mediation between COVID-19, its symptoms and a chain of effects that ran from tiredness to motivation, through attention to immediate and delayed memory. This made it the first Parallel Paths Model, which included both a chain of mechanistic relationships between dependent variables and a stepwise influence of COVID-19. While the influence of COVID-19 was treated independently for each dependent variable, a central role of tiredness and motivation in cognitive performance was retained.

**Model 4**, the second Parallel Paths Model, retained the direct mediation of COVID-19 and its features, but without the interdependence that was hypothesized to exist between attention and memory task accuracy.

The models were evaluated on six fit metrics:

- Akaike information criterion (AIC)
- Bayesian information criterion (BIC)
- Comparative Fit Index (CFI)
- Tucker–Lewis Index (TLI)
- Root Mean Squared Error Approximation (RMSEA)
- Standard Root Mean Residual (SRMR)

These metrics were included to balance baseline model and covariance similarity with penalizations for complexity. For example, BIC, TLI and RMSEA each penalize complexity while performing very similar calculations to AIC, CFI and SRMR, respectively (see Table 3 for fit results).

**Table 3:** Fit metrics for each of the four constructed models

Model	AIC	BIC	CFI	TLI	RMSEA	SRMR
Model 1 (HPM1)	27 431.79	**27 602.64**	0.9771	0.9688	0.0369	0.0422
Model 2 (HPM2)	27 423.41	27 631.63	0.9847	0.9695	0.0365	0.0331
Model 3 (PPM1)	**27 409**.**78**	27 665.05	**0**.**9964**	**0**.**9823**	**0**.**0278**	**0**.**0121**
Model 4 (PPM2)	27 424.61	27 680.88	0.9886	0.9432	0.0498	0.0257

Best fit scores for each metric are highlighted in bold font.

As a comparator to the COVID-19 model, we also conducted path analysis examining the associations of mental health disorders and typical ageing with the same set of fatigue, attention and memory features. The intention of this control step was to highlight paths that could support both the hypothesized mechanistic relationship between the dependent variables and the distinct pattern of effects specific to COVID-19, relative to other data captured in this dataset. GP diagnoses of anxiety and depression were included in one model, while the effect of ageing was removed from the nuisance variable model and added to its own structural equation model. Hence, the final step incorporated three different models that calculated paths for COVID-19, ageing and anxiety/depression, respectively, influencing the chain mechanism highlighted in the above analysis. Fit metrics were not employed for comparison here as the models were not nested (i.e. they relied on different variables for defining paths). Path coefficients were evaluated using Maximum Likelihood estimation, with non-parametric bootstrapping (5000 iterations) to derive standard errors and confidence intervals for all model parameters. Robust estimates were derived from bias-corrected bootstrapped confidence intervals (95% CI), in order to ensure that the rank-inverse transformation of the data did not lead to biased *P*-values.

## Results

### Cognitive performance and subjective fatigue related to COVID-19 symptom duration

Given that the present memory task data were taken from a much larger sample, simple bivariate regressions were used to confirm previous findings that immediate recall accuracy declined with longer COVID-19 duration (*P* < 0.001), with the strongest decrement observed in participants with unresolved symptoms (−0.27 SD). Among other previous observations, hospitalization (*P* < 0.001, −0.33 SD) and brain fog (*P*  *<* 0.001, −0.06 SD) both remained significant. These analyses replicate earlier reports from the larger dataset.^10^ All relevant statistics are reported in Section 3 of the Supplemental Data.

Analysis of variance for vigilance task accuracy across blocks revealed that any associations with hospitalization status and variant epoch were statistically non-significant, with a sub-threshold effect for participants who visited intensive care (*P* = 0.08). DVT accuracy did not decline across blocks, but rather increased (*F*(3, 24 080) = 5.26, *P* = 0.002) indicating a potential learning effect as participants became more familiar with the task requirements (Fig. 3). DVT accuracy was also associated with COVID-19 symptom duration (*F*(4, 24 080) = 7.56, *P* < 0.001). Relative to a no-COVID-19 baseline, participants with ongoing persistent symptoms demonstrated the largest deficits (−0.198 SD, *P* < 0.001). DVT reaction time was also related to symptom duration (*F*(4, 24 080) = 2.52, *P* = 0.038), with again slower responses in participants who experienced ongoing symptoms (0.107 SD, *P* = 0.037) (see Supplementary Tables 4–6 and 13–15).

**Figure 3:**
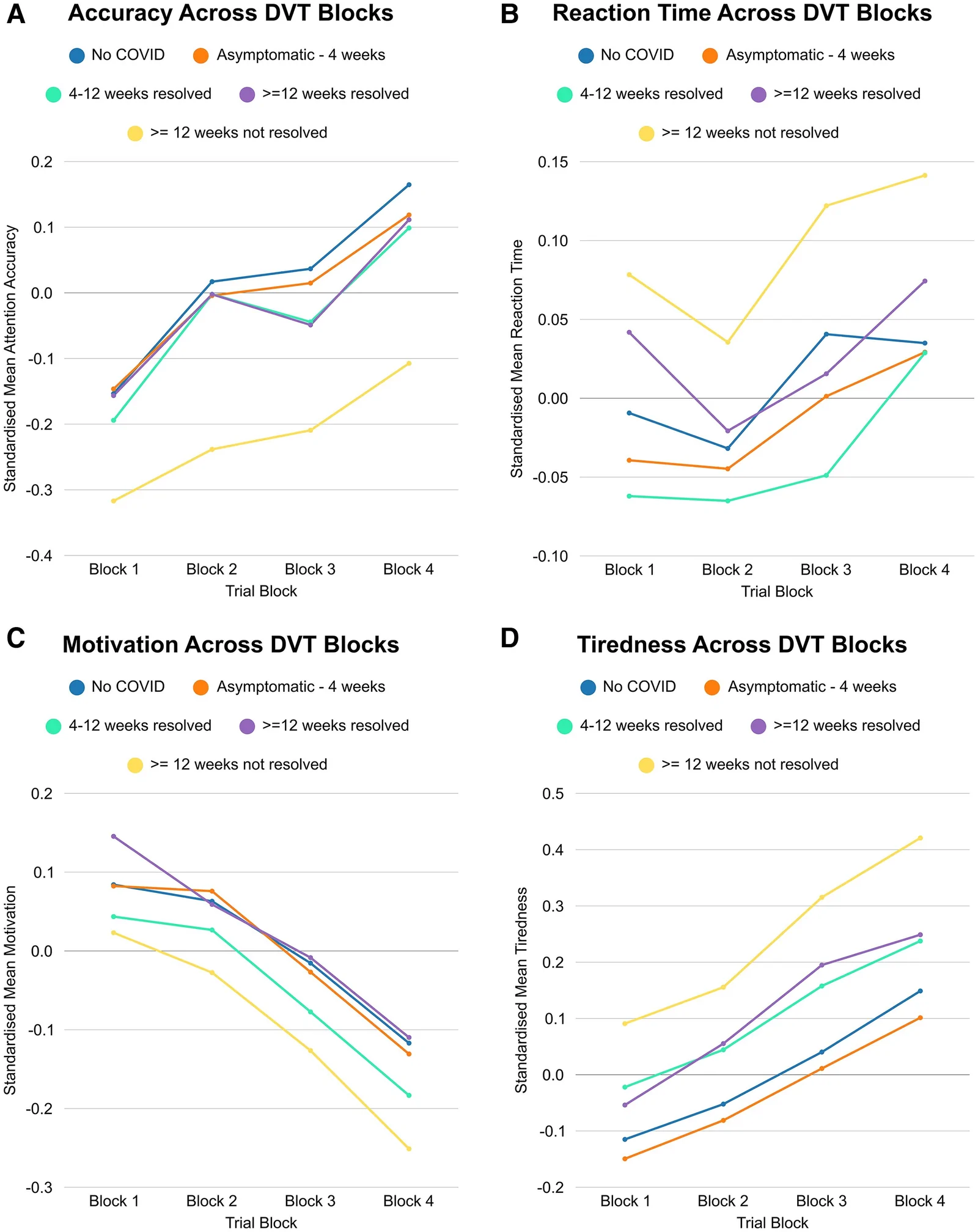
**Blockwise estimates for DVT measures.** Repeated measures ANOVA yielded standardized means across blocks of trials, with estimates of changes across blocks shown for **(A)** DVT accuracy, **(B)** reaction time, **(C)** motivation and **(D)** tiredness, split by COVID-19 symptom duration category. Participants who had unresolved symptoms beyond 12 weeks (yellow line, *N* = 548) showed worse performance on all metrics, although motivation was non-significant. Plots highlight **(A)** DVT accuracy block effect (*F*(3, 24 080) = 5.26, *P* = 0.002) and COVID-19 duration effect (*F*(4, 24 080) = 7.56, *P* < 0.001), **(B)** reaction time COVID-19 duration effect (*F*(4, 24 080) = 2.52, *P* = 0.038), **(C)** motivation block effect (*F*(3, 24 080) = 11.98, *P* < 0.001) and **(D)** tiredness block effect (*F*(3, 24 080) = 23.96, *P* < 0.001) and COVID-19 duration effect (*F*(4, 24 080) = 10.85, *P* < 0.001).

ANOVA results also indicated that tiredness increased between each block of trials (*F*(3, 24 080) = 23.96, *P* < 0.001), and was higher in participants who had been hospitalized (*F*(2, 24 080) = 5.91, *P* = 0.002) or admitted to intensive care (*F*(2, 27 564) = 3.76, *P* = 0.023). COVID-19 duration was also associated with tiredness (*F*(4, 24 080) = 10.85, *P* < 0.001), as those with persistent ongoing symptoms beyond 12 weeks reported higher tiredness (0.239 SD, *P* < 0.001). In contrast, motivation declined across blocks (*F*(3, 24 080) = 11.98, *P* < 0.0001) but did not differ by COVID-19 or hospitalization status, suggesting an overall increase in fatigue during the DVT task (see Supplementary Tables 7–12).

To consolidate these results and evaluate effect sizes, two linear regression models were applied to assess overall vigilance task performance as related to COVID-19 features, measured by DVT reaction time and DVT accuracy. The model for DVT accuracy (*F*(13, 24 081) = 588, *P* < 0.001, *R*^2^ = 0.240) showed that unresolved symptoms beyond 12 weeks were associated with lower DVT accuracy versus the no-COVID-19 reference (*β* = −0.21, *P* < 0.001), while higher DVT accuracy was also related to lower tiredness (*β* = −0.027, *P* < 0.001), higher motivation (*β* = 0.19, *P* < 0.001) and faster reaction times (*β* = 0.43, *P* < 0.001). For regression on reaction time, the model fit was robust (*F*(13, 24 081) = 467, *P* < 0.001, *R*^2^ = 0.201) and revealed associations with both DVT accuracy (*β* = −0.455, *P* < 0.001) and motivation (*β* = 0.038, *P* < 0.001), while those with COVID-19-specific variables were non-significant (see Fig. 4).

**Figure 4:**
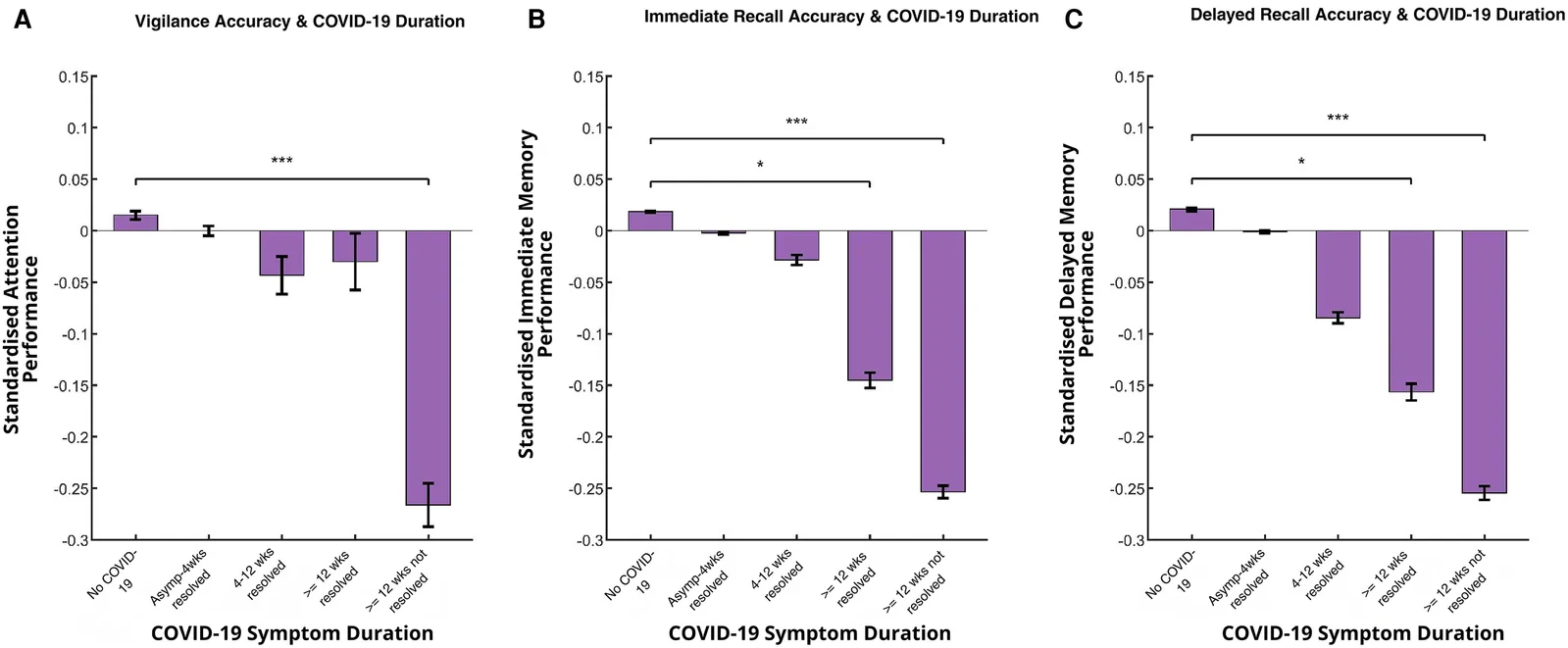
**Cognitive deficits and COVID-19 duration.** Plotted linear regression results produced standardized mean estimates for (**A**) DVT accuracy (*F*(13, 24 081) = 588, *P* < 0.001, *R*^2^ = 0.240), (**B**) immediate recall accuracy (*F*(17, 24 077) = 21.9, *P* < 0.001, *R*^2^ = 0.015) and (**C**) delayed recall accuracy (*F*(17, 24 077) = 27.3, *P* < 0.001, *R*^2^ = 0.018), separated for each COVID-19 duration category. Models were adjusted for age, sex, ethnicity, education, vaccination status and deprivation index, with scores standardized via inverse rank transformation. Adjustments made for comorbidities and corresponding results are available in Supplementary Data Section 1. Data points represent mean standard deviations in task accuracy for each represented group. Mean standard deviations were calculated for each participant comprising that group, with one value per participant for a total of 24 095 participants. Participant groups were composed of the following: No COVID-19: *N* = 12 170, asymptomatic—4weeks resolved: *N* = 10 386, 4–12 weeks resolved: *N* = 688, ≥12 weeks resolved: *N* = 303, ≥12 weeks not resolved: *N* = 548.

Two further linear regression models were used to evaluate how COVID-19 features and vigilance task data related to memory task performance. Unlike the DVT models, for which COVID-19 variant was not selected due to the absence of ANOVA results, both memory models contained COVID-19 variants as predictors due to their relevance in previous memory task analyses.^10^ For immediate memory, the model (*F*(17, 24 077) = 21.9, *P* < 0.001, *R*^2^ = 0.015) indicated that there were lower scores for unresolved symptoms beyond a 12-week period (*β* = −0.45, *P* < 0.001). Greater tiredness (*β* = −0.04, *P* < 0.001) and lower DVT accuracy (*β* = 0.10, *P* < 0.001) were also related to immediate memory scores, while motivation was non-significant.

The delayed memory model (*F*(17, 24 077) = 27.3, *P* < 0.001, *R*^2^ = 0.018) echoed these findings, as it was also associated with unresolved symptoms after 12 weeks (*β* = −0.36, *P* = 0.004), increased tiredness (*β* = −0.05, *P* < 0.001), and lower DVT accuracy (*β* = 0.11, *P* < 0.001). As with immediate memory, motivation remained non-significant. All other linear regression findings are included in Supplementary Tables 16–23 in the Supplemental Data.

### Interactions with COVID-19-related symptoms

Subjective symptom data included binary reports of brain fog and memory problems, as well as three levels of post-COVID-19 fatigue. Stepwise regression was used to identify parsimonious models that included interactions between cognitive performance, fatigue and symptoms.

For DVT accuracy, the selected model (*F*(5, 24 089) = 275, *P*  *<* 0.001, *R*^2^ = 0.0537) retained tiredness (*β* = −0.001, *P* = 0.006), motivation (*β* = 0.010, *P* < 0.001), brain fog (*β* = −0.067, *P* < 0.001), and an interaction between self-reported memory problems and tiredness (*β* = −0.002, *P* < 0.001) while memory-problem symptoms were retained but non-significant.

The immediate memory model (*F*(5, 24 089) = 76.2, *P* < 0.001, *R*^2^ = 0.0154) selected hospitalization (*β* = −0.283, *P* < 0.001), tiredness (*β* = −0.001, *P* < 0.001), DVT accuracy (*β* = 0.091, *P* < 0.001), memory-problem symptoms (*β* = −0.060, *P* < 0.001) and an interaction between DVT accuracy and memory problems (*β* = 0.073, *P*  *<* 0.001).

The delayed memory model (*F*(5, 24 089) = 90.4, *P* < 0.001, *R*^2^ = 0.0182) yielded the same factors as above: hospitalization (*β* = −0.32, *P* < 0.001), tiredness (*β* = −0.002, *P* < 0.001), DVT accuracy (*β* = 0.10, *P* < 0.001), memory-problem symptoms (*β* = −0.073, *P* < 0.001), and the DVT accuracy–memory problems interaction (*β* = 0.057, *P* = 0.001). Full stepwise results are available in Supplementary Tables 24–29.

### Parallel and hierarchical pathways between COVID-19 features, fatigue and cognitive performance

Results from the previous analyses informed the construction of four different path models (see Fig. 2). Fit indices for all tested models are presented in Table 3. Model 3 showed the best overall fit (AIC lowest; CFI = 0.996, TLI = 0.982, RMSEA = 0.028, SRMR = 0.012). The model contained a parallel relationship between COVID-19 features, subjective fatigue and cognitive performance, as well as hierarchical paths between the constituent elements of fatigue and cognitive performance.

Its BIC was slightly higher than the strictly hierarchical Model 1 (ΔBIC = 63), reflecting greater complexity, but improvements on other indices were substantial. Model 3 also had the most favourable χ^2^ (χ^2^(6) = 13.14, *P* = 0.041). Remaining models showed poorer fit (*P* < 0.001). Model 1 achieved the lowest BIC due to parsimony, since it only contained a direct path between COVID-19 features and tiredness, but underperformed elsewhere.

Model 3 was estimated with 5000 bootstrap resamples for 95% CIs. Regression statistics for model paths were provided to identify significant relationships between each of the variables that composed the structured model. DVT tiredness was found to be inversely related to reported motivation (*β* = −0.381, SE = 0.031, *P* < 0.001, 95% CI [−0.441, −0.321]), which in turn was positively related to DVT accuracy (*β* = 0.234, SE = 0.025, *P* < 0.001, 95% CI [0.183, 0.282]). DVT accuracy was also associated with immediate recall (*β* = 0.121, SE = 0.027, *P* < 0.001, 95% CI [0.069, 0.173]), and delayed recall was strongly related to immediate recall (*β* = 0.775, SE = 0.015, *P* < 0.001, 95% CI [0.746, 0.804]).

In terms of parallel relationships between cognitive performance and COVID-19, there were direct paths from COVID-19 symptom duration to both DVT accuracy (*β* = −0.082, SE = 0.028, *P* = 0.003, 95% CI [−0.136, −0.026]) and immediate recall (*β* = −0.081, SE = 0.028, *P* = 0.006, 95% CI [−0.138, −0.024]). There was also an indirect path from COVID-19 duration through DVT accuracy to delayed recall, suggesting a hierarchical relationship within the parallel paths model (*β* = −0.064, SE = 0.022, *P* = 0.003, 95% CI [−0.105, −0.020]).

For immediate recall, we observed a direct path to hospitalization status following COVID-19 (*β* = −0.268, SE = 0.104, *P* = 0.010, 95% CI [−0.471, −0.068]). Tiredness during the DVT was higher for participants with reported brain fog (*β* = 0.267, SE = 0.066, *P* < 0.001, 95% CI [0.136, 0.396]. Although the direct path between brain fog and DVT accuracy fell short of significance (*β* = −0.107, SE = 0.063, *P* = 0.090, 95% CI [−0.230, 0.014]), there was an additional indirect path from brain fog through tiredness downstream to all other fatigue and cognitive performance measures (*β* = −0.002, SE = 0.001, *P* = 0.006, 95% CI [−0.004, −0.001]) (see Fig. 5 for direct path coefficients).

**Figure 5:**
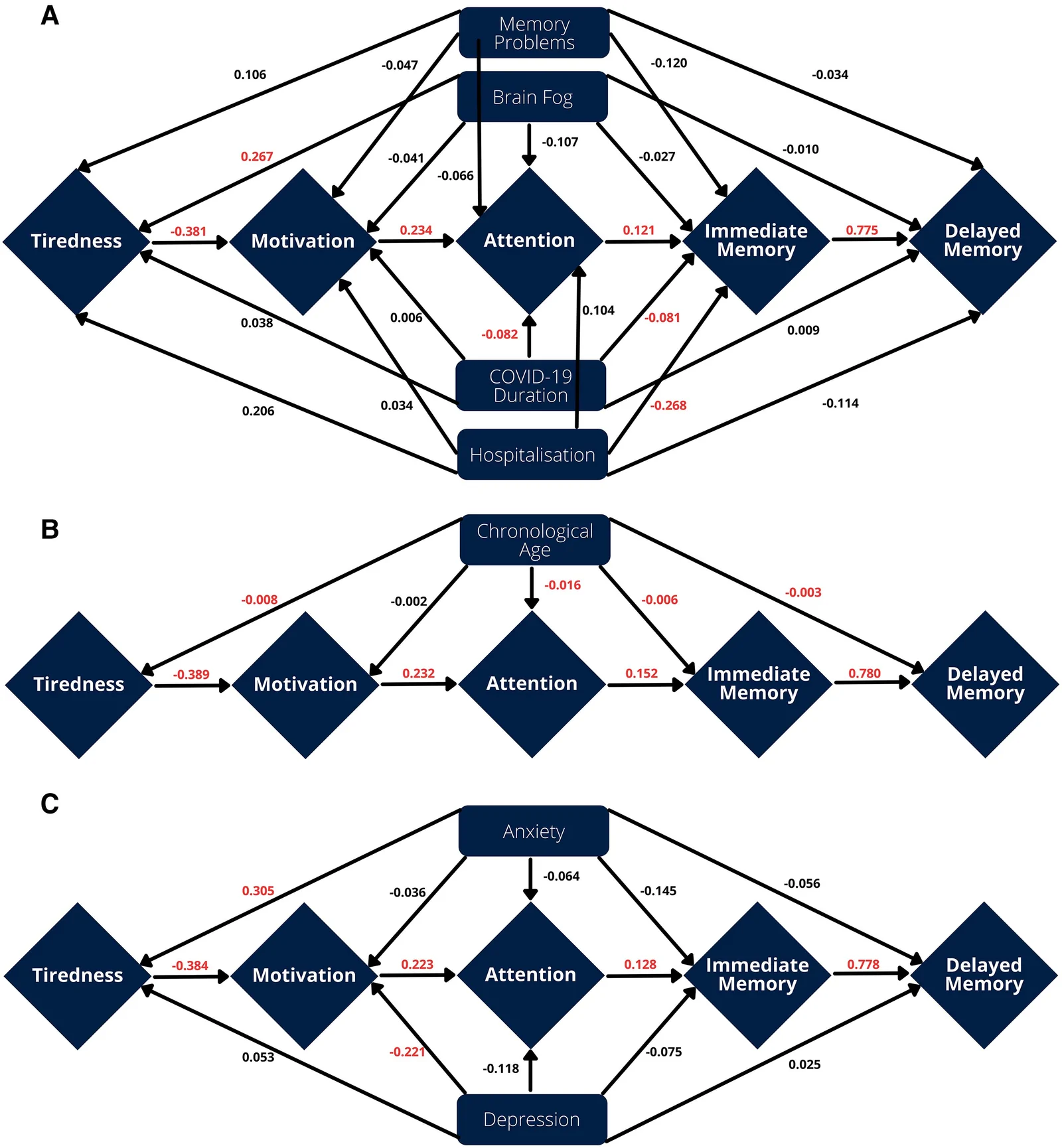
**Compared path models.** Path regression coefficients were estimated using Maximum Likelihood estimation. Non-parametric bootstrapping (5000 iterations) was used to derive bias-corrected 95% confidence intervals for all model parameters for *N* = 24 095. Direct paths between COVID-19 and cognition are shown in the final model **(A)**, where COVID-19 duration, severity and symptomatology are related to dependent variables in parallel. The model for ageing **(B)** exhibits somewhat weaker effects compared to strong interdependence of cognitive functions, while the model for anxiety and depression **(C)** demonstrates direct paths to tiredness and motivation, respectively, with no direct effect on cognition. All significant paths are highlighted in red.

For effect size, *R*^2^ was calculated for Tiredness (0.034), Motivation (0.153), DVT accuracy (0.075), Immediate Memory (0.034) and Delayed Memory (0.636). See Supplementary Fig. 3 in Supplemental Data for model specification and Supplementary Tables 30–39 for full path analysis results.

Control models were structured to evaluate the proposed relationship between fatigue and cognitive performance as associated with normal ageing and mental health records that were present in the dataset.

Normal ageing yielded a strong model fit χ^2^(6) = 16.826, *P* = 0.010; CFI = 0.995, TLI = 0.987, RMSEA = 0.034, SRMR = 0.018. Age related negatively to tiredness (*β* = −0.008, SE = 0.002, *P* < 0.001, 95% CI [−0.012, −0.004]) and was also associated with a decrease in DVT accuracy (*β* = −0.016, SE = 0.002, *P* < 0.001, 95% CI [−0.019, −0.0.012]). Again, there was a strong DVT accuracy to immediate memory path relationship (*β* = 0.141, SE = 0.027, *P* < 0.001, 95% CI [0.087, 0.195]), while direct paths also existed from age to immediate memory (*β* = −0.006, SE = 0.002, *P* = 0.002, 95% CI [−0.010, −0.002]) and delayed memory (*β* = −0.003, SE = 0.001, *P* = 0.019, 95% CI [−0.005, −0.000]). There was also an indirect age-to-memory path via tiredness (*β* = −0.006, SE = 0.002, *P* < 0.001, 95% CI [−0.009, −0.003]).

The second control model used GP diagnoses of anxiety and depression as predictors for changes in fatigue and cognitive performance. Model fit was again robust (χ^2^(6) = 9.956, *P* = 0.020; CFI = 0.99, TLI = 0.98, RMSEA = 0.031, SRMR = 0.017). There was a direct path from anxiety to tiredness (*β* = 0.305, SE = 0.092, *P* = 0.001, 95% CI [0.122, 0.479]) and (negatively) for depression with motivation (*β* = −0.221, SE = 0.070, *P* = 0.002, 95% CI [−0.356, −0.083]). No other direct paths to objective cognitive measures were significant. An indirect path linked anxiety to immediate memory via tiredness (*β* = 0.237, SE = 0.072, *P* = 0.001, 95% CI [0.095, 0.376]) while there was no analogous path for motivation. Direct paths for all three models are highlighted in Fig. 5.

## Discussion

We used data from the REACT-LC cohort to examine relationships between COVID-19, tiredness, motivation, attention and memory at a large population scale. Our findings confirm that individuals with persistent symptoms post-COVID-19 show deficits in both attention and memory compared to those with no history of infection, consistent with previous reports of post-COVID cognitive impairment.^10,20,22,23,26,59^ Self-reported tiredness and reduced motivation during task performance were associated with lower attention and memory accuracy, indicating that fatigue, as defined by both tiredness and motivation, is one determinant of objective cognitive performance. This finding aligns with previous research linking fatigue to sustained attention.^35-37,39-43^

Path analysis supported a model in which COVID-19 symptom duration was associated with cognitive performance through multiple direct and indirect routes. The best-fitting structure (Model 3) incorporated both hierarchical and parallel paths: attention and memory performance were directly related to COVID-19 duration and hospitalization status in parallel, while brain fog was linked to tiredness which had a downstream association with cognitive performance, representing a potential hierarchical mechanism (Fig. 5). This model provided the strongest overall fit and was consistent with the hypothesis that Long COVID has a multi-faceted impact on cognition, involving both fatigue-driven and domain-specific mechanisms. While a large proportion of delayed memory task accuracy variance was explained by this model (*R*^2^ = 0.636) there were comparatively smaller effects observed for the other variables. In addition, the SD unit differences in DVT accuracy (−0.082) and immediate memory accuracy (−0.081) related to COVID-19 duration were also small, but their inclusion improved the model fit, suggesting their relevance to relationships between Long COVID and cognitive deficits. Furthermore, the observed directionality from fatigue through to cognitive performance could accord with a hierarchical influence of arousal and resource allocation on higher cognitive functions, consistent with frameworks proposing that fatigue impairs cognitive control.^39,40,42,60,61^ At the same time, persistent COVID-19 symptoms are shown here to have parallel associations with distinct cognitive systems, producing a hybrid architecture that combines hierarchical and parallel mechanisms. This pattern accords with neurobiological models implicating widespread network hypoarousal or neuroinflammatory disruption following infection.^3-8,61,62^

Subjective reports of brain fog, a frequent long-COVID symptom,^20-30^ were directly associated with tiredness and indirectly related to cognitive performance. This evidence suggests that brain fog may be characterized as a fatigue-driven symptom which impacts attentional performance and by extension disrupts encoding of memories for attended stimuli. This also indicates that brain fog is not an isolated memory issue, consistent with findings from other post-COVID-19 cohorts,^23,26,32,34^ instead suggesting that cognitive complaints in Long COVID may be partly explained both by arousal dysregulation and issues with attentional vigilance.

While subjective fatigue symptoms and objective cognitive performance were related, the strengths of these associations were modest, consistent with prior findings.^23-29,33,34^ This implies that subjective cognitive complaints are shaped both by interoceptive factors, in this case reports of fatigue and brain fog, and by measurable cognitive performance. Therefore, both forms of measurement capture important but distinct facets of post-COVID-19 cognitive deficits. Objective tasks quantify performance limitations that may affect everyday functioning even when unrecognized by individuals, whereas subjective complaints reflect perceived effort, fatigue, and interoceptive awareness of these experiences. Integrating both perspectives provides a more complete account of cognitive status and functional impact in Long COVID.

Deficits in attention and memory accuracy were smaller in magnitude yet still detectable for patients who had recovered from Long COVID, consistent with residual effects after clinical recovery. While this graded pattern suggests partial restoration of cognitive function over time, it also indicates that the impact of Long COVID may persist beyond symptom resolution for some individuals. However, this should not be considered a completely continuous effect, since the direct relationship to attention performance may be primarily driven by the difference between those who were still suffering from Long COVID at the time of testing and those who had never been infected. Nonetheless, it is demonstrated here that there exists a graded linear relationship between COVID-19 symptom duration and memory performance. This indicates residual impairments may attenuate depending on symptom duration, as has been reported in other large cohorts,^10,12,23,26,59^ implying that neural or metabolic mechanisms underlying fatigue and attentional control may recover slowly or incompletely. These observations reinforce the view that Long COVID exerts both transient and potentially enduring influences on cognitive networks, depending on severity, symptom duration, and individual resilience factors. Longitudinal research is required to confirm this hypothesis.

Comparison of different path models highlighted the distinctive cognitive profile of Long COVID. The ageing model produced a similar structure but with weaker relationships: ageing was associated with lower reports of on-task fatigue as well as lower attention and memory accuracy, with the latter result indicative of age-related reductions in cognitive performance rather than specific post-viral deficits.^60^ By contrast, the model of anxiety and depression revealed associations of anxiety with tiredness (direct) and depression with motivation (inverse), although neither disorder showed direct paths to attention or memory accuracy.^51,52^ Indirect effects were limited to downstream relationships mediated through tiredness. These findings align with prior studies showing that mood and anxiety influence subjective fatigue but not objective cognitive measures.^23,24,29^ Together, these comparisons suggest that Long COVID involves a more distributed constellation of interdependent effects, combining the fatigue pathways typically seen in mental-health conditions with additional direct impairments in attention and memory, possibly reflecting neurobiological disruption beyond that seen in normal ageing or mood disorders.

Our study has limitations. This was a cross-sectional analysis: structural equation models infer associations rather than causal relationships, and apparent mediation cannot confirm temporal ordering.^63^ It is worth noting that, although results were age adjusted, there is a potential for reverse causal relationship where a COVID-19-related cognitive deficits may demask pre-existing cognitive deficits in older individuals with subclinical neurodegenerative disorders. Longitudinal studies using repeated cognitive assessments would help to verify this directionality, additionally testing whether attentional deficits precede memory impairment or evolve in parallel. Although the REACT-LC sample is large and demographically diverse, participation was voluntary and online, raising the possibility of selection bias and self-report error. Our DVT was short compared to more common sustained-attention paradigms as it was included in a battery comprising surveys and eight other tasks. Although it proved to be amongst the most sensitive tasks to post-COVID-19 cognitive deficits in this study, an increase in accuracy across blocks was observed, which likely reflects a learning effect. Extended testing might reveal stronger time-on-task declines, especially in those with long COVID, given previous reports of accelerated vigilance decrements when applying longer protocols in previous studies.^20,39^

Neurobiological mechanisms such as inflammation, vascular changes, or altered functional connectivity were not directly measured; future multimodal studies combining cognitive modelling with neuroimaging or physiological markers (e.g. 3, 64, 65) are needed to elucidate the neural substrates of these pathways. Future work should also test whether interventions targeting attentional control or fatigue (e.g. arousal modulation, cognitive pacing, motivational training) can improve performance, and whether sustained-attention metrics can serve as sensitive endpoints in clinical trials for Long COVID or other post-viral conditions. In addition, since cognitive symptoms appear more commonly for COVID-19 patients compared to those with influenza,^64^ further studies should include case-control groups to understand the nuanced differences between COVID-19 sequelae and those of other viral infections.

Clinically, our findings suggest that sustained-attention impairment may represent a core neurocognitive feature of Long COVID, contributing to the memory performance deficits and more modestly to subjective cognitive symptoms. Tiredness and motivational depletion appear to act as proximal modulators of this attentional deficit, although it is the former rather than the latter that seems the more prominent feature in people with ongoing persistent symptoms post-COVID-19. Recognizing this hierarchy may help refine diagnostic assessments, guiding clinicians towards tasks that index attentional vigilance rather than relying only on self-report. Importantly, subjective and objective measures should be viewed as complementary rather than interchangeable. Weak statistical correlations between them do not diminish their joint clinical relevance: subjective complaints may guide symptom management and rehabilitation priorities, while objective tests delineate the cognitive domains most affected. Together, they provide a fuller understanding of functional capacity and quality of life in patients recovering from COVID-19. From a theoretical standpoint, the results support a multi-system model in which post-viral cognitive dysfunction reflects both domain-general arousal dysregulation and domain-specific cognitive compromise. The concurrent hierarchical and parallel architecture of effects, distinct from that seen in normal ageing or mood disorders, suggests that COVID-19-related neurobiological changes may disrupt multiple cognitive-control and memory systems simultaneously. Integrating these insights with emerging neuroimaging evidence of cortical hypoarousal and hippocampal dysfunction^3,61,62^ could advance mechanistic understanding and inform rehabilitative strategies.

In summary, our modelling framework provides a foundation for linking subjective symptoms such as brain fog and fatigue with objective cognitive performance measures. Such integration may help streamline cognitive screening in post-COVID-19 clinics and guide future interventions targeting fatigue–attention–memory interactions that are prominent in Long COVID.

## Supplementary Material

fcag361_Supplementary_Data

## Data Availability

The REACT-LC data used in this study are held by Imperial College London. Access to this data may be made available to researchers subject to a data access agreement. Enquiries should be directed to the corresponding author. R analysis scripts for path analyses reported in this study are available from the corresponding author upon reasonable request.
