# Supplementary material for: A cognitive architecture for Long COVID: the interplay of brain fog, fatigue, attention and memory
Source: Brain Commun. 2026 Sep 24;8(5):fcag361. doi: 10.1093/braincomms/fcag361 (PMC13644365; doi:10.1093/braincomms/fcag361)
Supplement: Supplementary file 1 [file fcag361_supplementary_data.pdf]

Supplemental Data

Section 1 contains a sensitivity analysis to confirm the development of our regression models.

Section 2 contains distribution plots for age and COVID-19 categories.

Section 3 contains results tables presented for each step of the analysis, with distribution plots for main dependent variables across COVID-19 and Age Groups. Model specification code is also provided for the final selected path model (PPM1).

1. Sensitivity Analysis

Our previous regression models were developed after residualising task scores following confound adjustment for demographic variables. It was prudent to also evaluate the models for sensitivity by including extensive comorbidity information attached to the REACT dataset, which included both self-reports and electronic health records. In all, there were 45 different conditions, although some of these included 'other' categories. A full list of comorbidities is included below.

The analysis (see Supplemental tables i-iii) indicates that there was a negligible material difference to our outcome variables when these 45 conditions were included as confounds. In other words, it is likely that demographic variables associated with these comorbidities accounted for much of the age-related comorbidities influence on the model's outcomes, while low n for many serious conditions meant they played a minimal role.

Below, adjusted results are presented for our three primary outcome variables (DVT accuracy, immediate memory accuracy and delayed memory accuracy). The inclusion of the extra comorbidity confounds led to negligible changes to adjusted R^2 values, indicating that they were not responsible for changes to effect sizes. Original DVT analysis: $R^2 = 0.240$ vs Sensitivity Analysis: $R^2 = 0.239$; Original Immediate Memory Analysis: $R^2 = 0.015$ vs Sensitivity Analysis: $R^2 = 0.0145$; Original Delayed Memory Analysis: $R^2 = 0.018$ vs Sensitivity Analysis $R^2 = 0.0178$. Sensitivity analysis regression tables are available below.

Supplementary Table 1: DVT Accuracy Sensitivity Analysis – Linear Regression.

Variable	Estimate	SE	tStat	pValue	CI_Lower	CI_Upper
(Intercept)	0.020882	0.019271	1.0836	0.27855	-0.01689	0.058654
Hospital_1_Yes	0.0042793	0.07446	0.057471	0.95417	-0.14167	0.15023
Hospital_1_unknown	-0.049243	0.1915	-0.25715	0.79707	-0.42459	0.32611
Intensive_care_1_Yes	-0.17602	0.13425	-1.3111	0.18983	-0.43915	0.087117
Intensive_care_1_unknown	0.14414	0.20855	0.69115	0.48948	-0.26463	0.5529
AandE_1_Yes	0.023219	0.075278	0.30844	0.75775	-0.12433	0.17077
AandE_1_unknown	-0.094724	0.13768	-0.68799	0.49146	-0.36459	0.17514
CAT_12_4-12 wks resolved	-0.058538	0.03872	-1.5118	0.13059	-0.13443	0.017356
**CAT_12_>=12 not resolved**	**-0.20629**	**0.042445**	**-4.8602**	**1.1801e-06**	**-0.28948**	**-0.1231**
CAT_12_>=12 resolved	-0.02641	0.053877	-0.49019	0.624	-0.13201	0.079192
CAT_12_Asymp-4wks resolved	-0.032296	0.020094	-1.6072	0.10802	-0.071682	0.0070897
**Tiredness**	**-0.024699**	**0.0060687**	**-4.0699**	**4.7183e-05**	**-0.036594**	**-0.012804**
**Motivation**	**0.18586**	**0.0060652**	**30.643**	**2.5256e-202**	**0.17397**	**0.19774**
**RT**	**-0.43271**	**0.0056391**	**-76.733**	**0**	**-0.44376**	**-0.42166**

Supplementary Table 2: Immediate Memory Sensitivity Analysis – Linear Regression

Variable	Estimate	SE	tStat	pValue	CI_Lower	CI_Upper
(Intercept)	0.020063	0.0219	0.91612	0.35961	-0.022862	0.062988
**Hospital_1_Yes**	**-0.2221**	**0.084767**	**-2.6202**	**0.0087943**	**-0.38825**	**-0.055955**
Hospital_1_unknown	0.27743	0.21743	1.276	0.20198	-0.14874	0.70359
Intensive_care_1_Yes	-0.15328	0.15262	-1.0044	0.31522	-0.45242	0.14586
Intensive_care_1_unknown	-0.14692	0.23678	-0.6205	0.53494	-0.61103	0.31719
AandE_1_Yes	-0.006992	0.085598	-0.081684	0.9349	-0.17477	0.16078
AandE_1_unknown	-0.13168	0.15632	-0.84235	0.3996	-0.43808	0.17472
variant_1_Alpha	0.13321	0.1226	1.0865	0.27725	-0.1071	0.37352
variant_1_Delta	0.1417	0.11989	1.1819	0.23725	-0.093291	0.37669
variant_1_Omicron	0.20603	0.11908	1.7302	0.083615	-0.027378	0.43944
variant_1_Wild	0.14274	0.12172	1.1727	0.24094	-0.095842	0.38132
CAT_12_4-12 wks resolved	-0.23916	0.12663	-1.8886	0.058962	-0.48737	0.0090541
**CAT_12_>=12 not resolved**	**-0.36091**	**0.12834**	**-2.8121**	**0.0049257**	**-0.61247**	**-0.10935**
**CAT_12_>=12 resolved**	**-0.29801**	**0.13417**	**-2.2211**	**0.026351**	**-0.56099**	**-0.035029**
CAT_12_Asymp-4wks resolved	-0.19841	0.12002	-1.6532	0.098309	-0.43366	0.036833

**Tiredness**	**-0.03949**	**0.006901**	**-5.7224**	**1.0627e-08**	**-0.053016**	**-0.025964**
**Attention**	**0.11032**	**0.0065692**	**16.794**	**6.1782e-63**	**0.097446**	**0.1232**
Motivation	0.003622	0.0070241	0.51565	0.60611	-0.010146	0.01739

Supplementary Table 3: Delayed Memory Sensitivity Analysis – Linear Regression

Variable	Estimate	SE	tStat	pValue
(Intercept)	0.021077	0.021937	0.9608	0.33666
**Hospital_1_Yes**	**-0.19304**	**0.084909**	**-2.2735**	**0.023008**
Hospital_1_unknown	0.20986	0.21779	0.96357	0.33527
Intensive_care_1_Yes	-0.085561	0.15287	-0.55969	0.5757
Intensive_care_1_unknown	-0.09892	0.23718	-0.41707	0.67663
AandE_1_Yes	-0.018967	0.085741	-0.22121	0.82493
AandE_1_unknown	-0.11588	0.15659	-0.74007	0.45926
variant_1_Alpha	0.2097	0.12281	1.7075	0.087738
variant_1_Delta	0.21764	0.12009	1.8123	0.069954
**variant_1_Omicron**	**0.27137**	**0.11928**	**2.275**	**0.022915**
variant_1_Wild	0.23586	0.12192	1.9345	0.053069
**CAT_12_4-12 wks resolved**	**-0.26178**	**0.12685**	**-2.0638**	**0.039048**
**CAT_12_>=12 not resolved**	**-0.44588**	**0.12856**	**-3.4684**	**0.00052458**
**CAT_12_>=12 resolved**	**-0.3736**	**0.1344**	**-2.7799**	**0.0054423**
**CAT_12_Asymp-4wks resolved**	**-0.2715**	**0.12022**	**-2.2583**	**0.023933**
**Tiredness**	**-0.034023**	**0.0069126**	**-4.9219**	**8.6266e-07**
**Attention**	**0.10253**	**0.0065802**	**15.582**	**1.7848e-54**
Motivation	-0.0019318	0.0070359	-0.27456	0.78366

List of Added Comorbidities:

Electronic Health Record Conditions:

Anxiety N = 1618

Depression N = 2614

Hypothyroid N = 1216

Stroke N = 113

Dementia N = 7

Rheumatoid Arthritis N = 224

Cancer (any)	N = 1045
Cancer (breast)	N = 436
Cancer (bladder)	N = 53
Cancer(gynae)	N = 67
Cancer (lowerGI)	N = 124
Cancer (prostate)	N = 286
Cancer (other)	N = 137
Hypertension	N = 4665
Diabetes	N = 1336
Asthma	N = 3125
Epilepsy	N = 185
Chronic Infection	N = 128

Self-reported conditions:

Organ transplant	N = 25
Diabetes	N = 780
Heart disease	N = 555
Hypertension	N = 2581
Stroke	N = 120

Kidney Failure N = 117

Liver disease N = 91

Anemia N = 304

Allergies N = 3100

Asthma N = 1314

Other lung disease N = 366

Cancer N = 460

Fibromyalgia N = 270

Epilepsy N = 87

Other brain/nerve condition N = 164

Osteoarthritis N = 1665

Immunosuppression N = 719

Chronic infection N = 27

Dementia N = 7

Anxiety N = 2416

Depression N = 1407

Other psychiatric disease N = 136

Rheumatoid arthritis N = 426

Hypothyroid	N = 813
Adrenal gland	N = 13
Sleep apnoea/ narcolepsy	N = 311
Other serious illness	N = 1031

2. Additional Figures

A

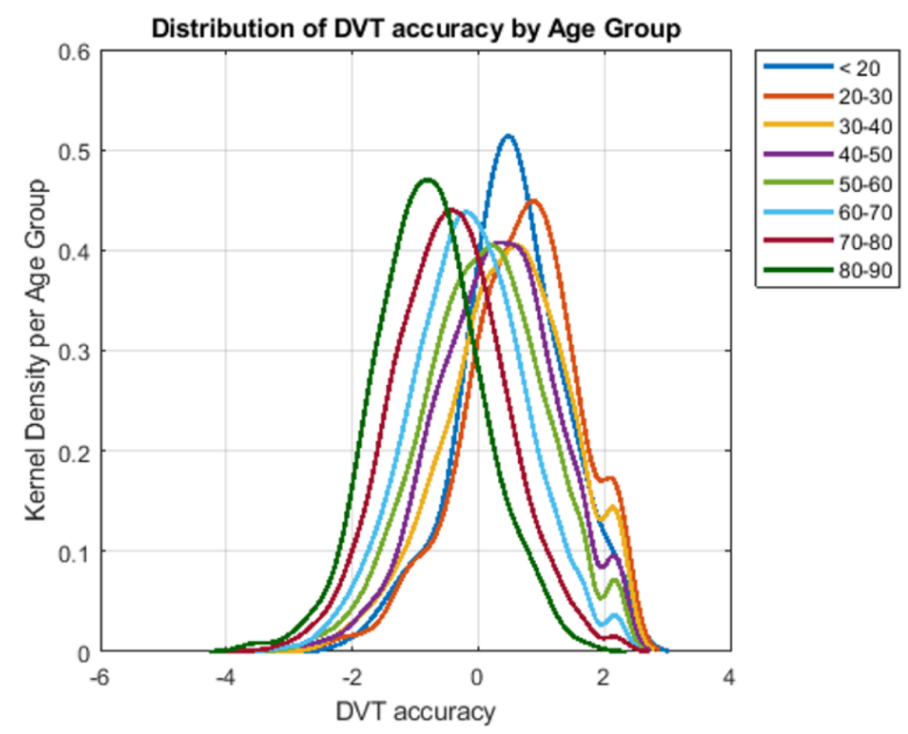

B

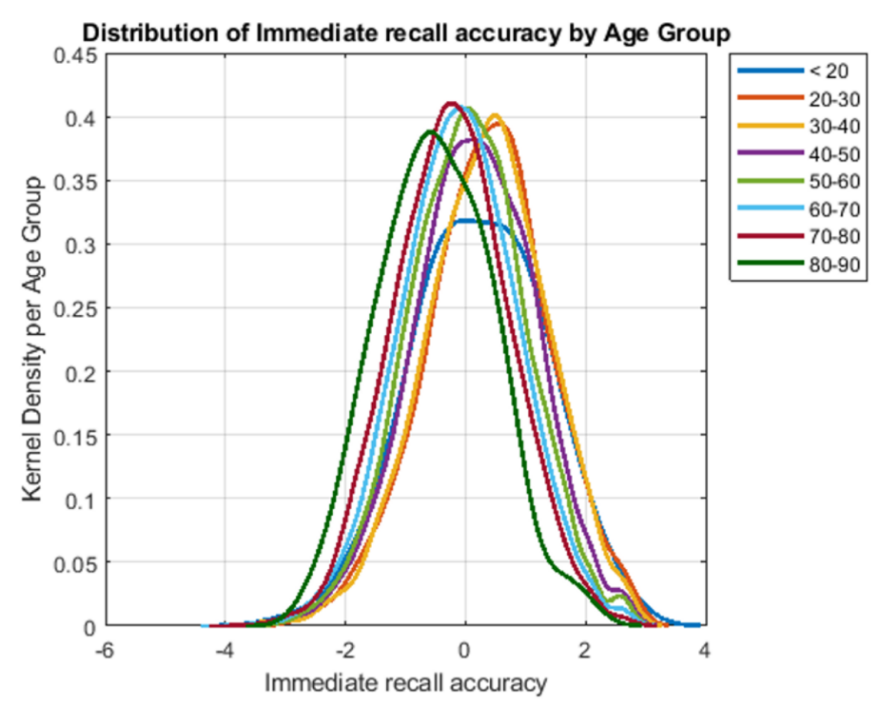

C

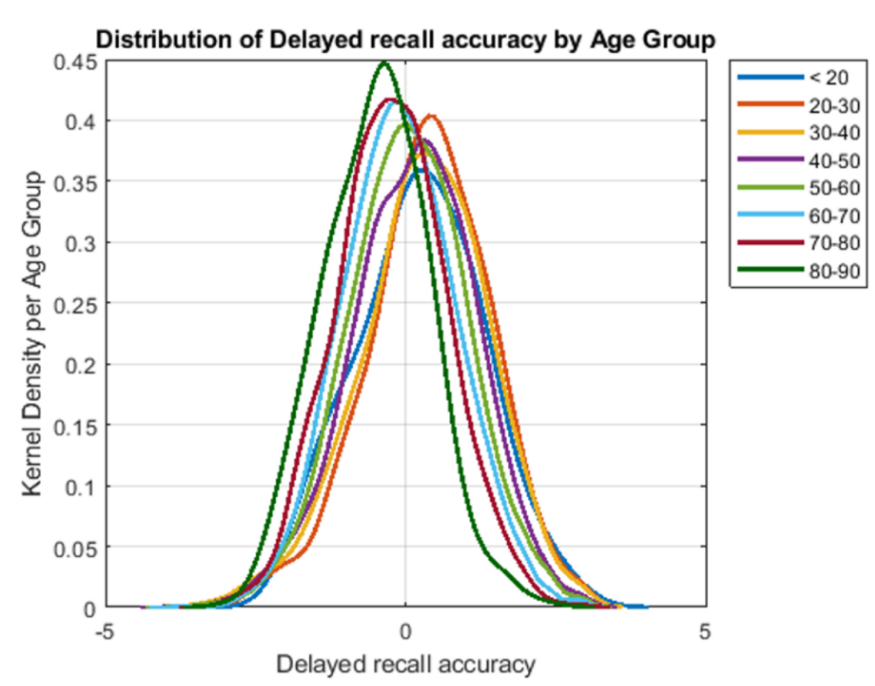

Supplementary Figure 1: DVT & memory accuracy across age groups. Kernel density estimation plots showing the distribution of (**A)** DVT accuracy, (**B**) Immediate memory accuracy and (**C)** Delayed memory accuracy for eight age groups. The x-axis represents cognitive performance, while the y-axis shows the probability density for each age group. The distributions reveal age-related differences in performance accuracy, with younger age groups (< 20 and 20–30 years) exhibiting peaks at higher accuracy values (~0.5), while older age groups progressively shift toward lower accuracy values. These patterns indicate a pattern of cognitive performance

typical of ageing. Age groups contained the following participant numbers: < 20: N = 202, 20-30: N = 970, 30-40: N = 2021, 40-50: N = 3458, 50-60: N = 6009, 60-70: N = 7054, 70-80: N = 3927, 80-90: N = 446.

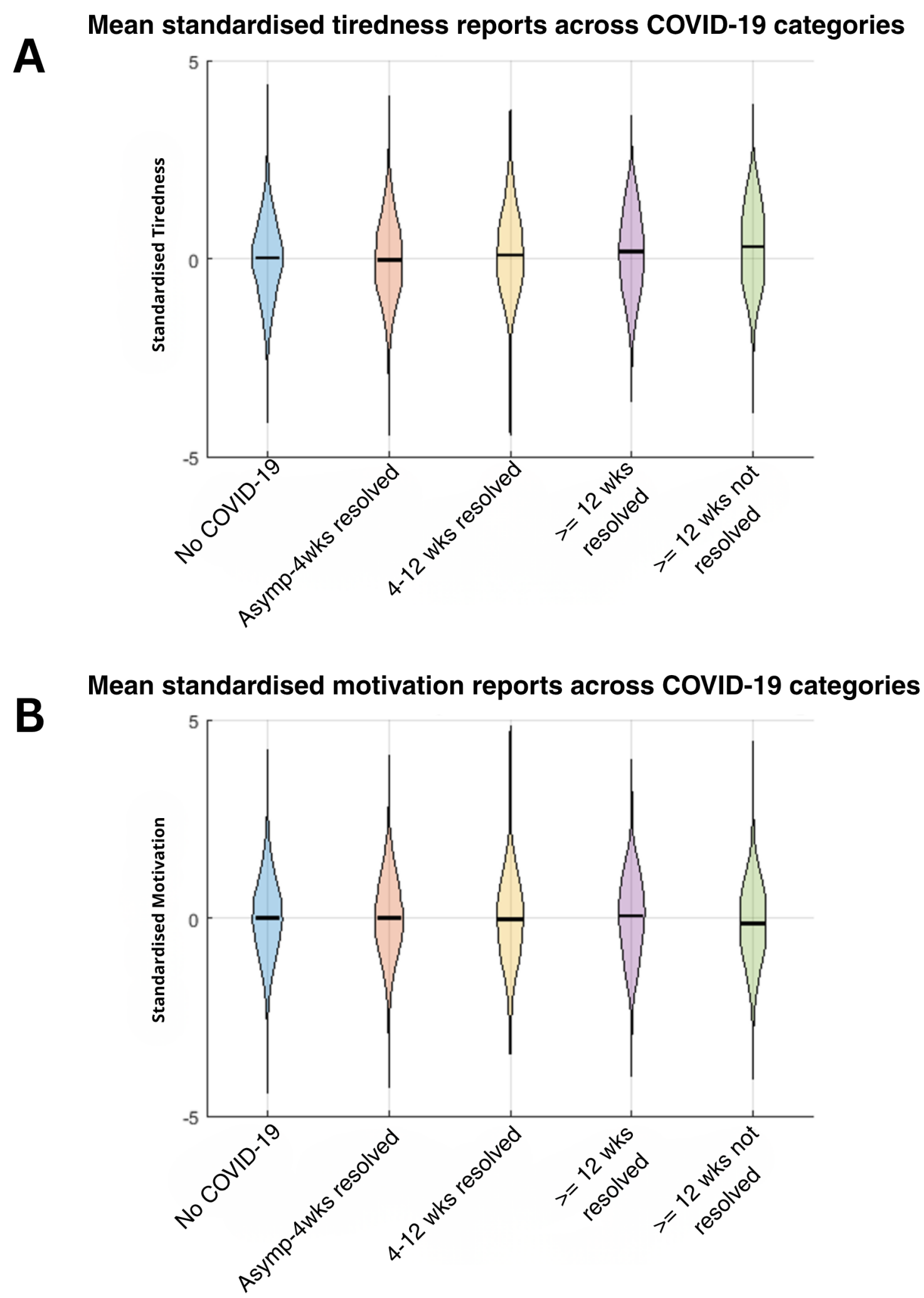

Supplementary Figure 2: Mean standardised tiredness and motivation across COVID-19 groups. Violin plots displaying the distribution of standardized scores for (**A**) tiredness and (**B**) motivation, as reported during the DVT task, by COVID-19 disease duration. Each violin width represents the probability density at each standardized value, with the white dot indicating the median and the thick black line showing the interquartile range. The distributions reveal comparable median motivation levels across all groups, though individuals with unresolved COVID symptoms show a notably wider distribution, suggesting greater heterogeneity of outcomes for this subgroup. These findings suggest that COVID-19 disease duration and resolution status have modest impact on population-level tiredness and motivation reports. Groups

represented were comprised of No COVID-19: N = 12170, Asymptomatic-4 weeks resolved: N = 10386, 4-12 weeks resolved: N = 688, >=12 weeks resolved: N = 303, >=12 weeks not resolved: N = 548.

3. Additional Results Tables

Repeated Measures ANOVA

Supplementary Table 4: DVT Accuracy – Repeated Measures (Main Effects)

Source	SumSq	DF	MeanSq	F	pValue	pValueGG	pValueHF	pValueLB
(Intercept)	28.426	1	28.426	10.931	0.00094725	0.00094725	0.00094725	0.00094725
Hospitalisation	3.6924	2	1.8462	0.70992	0.4917	0.4917	0.4917	0.4917
Intensive Care	13.064	2	6.5322	2.5118	0.08114	0.08114	0.08114	0.08114
AandE	0.89571	2	0.44786	0.17221	0.8418	0.8418	0.8418	0.8418
Variant	13.254	4	3.3134	1.2741	0.27758	0.27758	0.27758	0.27758
**COVID Duration**	**78.673**	**4**	**19.668**	**7.5631**	**4.3848e-06**	**4.3848e-06**	**4.3848e-06**	**4.3848e-06**
Error	62622	24080	2.6006					

Supplementary Table 5: DVT Accuracy – Repeated Measures (Block Effects)

Source	SumSq	DF	MeanSq	F	pValue	pValueGG	pValueHF	pValueLB
**Block**	**7.1045**	**3**	**2.3682**	**5.2618**	**0.0012558**	**0.0016051**	**0.0016043**	**0.021807**
Hospitalisation:Block	3.6102	6	0.6017	1.3369	0.23655	0.23957	0.23957	0.26268
Intensive Care:Block	3.1903	6	0.53172	1.1814	0.31276	0.31378	0.31377	0.30686
AandE:Block	4.746	6	0.791	1.7575	0.10352	0.10828	0.10827	0.1725
Variant:Block	4.103	12	0.34192	0.7597	0.69295	0.68475	0.68477	0.55136
COVID Duration:Block	4.9701	12	0.41418	0.92024	0.52525	0.52122	0.52123	0.45092
Error(Block)	32513	72240	0.45007					

Supplementary Table 6: DVT Accuracy – COVID Pairwise Comparisons

Group 1	Group 2	Difference	StdErr	pValue	Lower 95% CI	Upper 95% CI
no COVID	4-12 wks resolved	0.083927	0.10306	0.92632	-0.19719	0.36504
no COVID	>=12 not resolved	0.25702	0.10443	0.09959	-0.027837	0.54188
no COVID	>=12 resolved	0.06405	0.10919	0.97711	-0.2338	0.3619
no COVID	Asymp-4wks resolved	-0.058542	0.097678	0.97522	-0.2079	0.32499
4-12 wks resolved	no COVID	-0.083927	0.10306	0.92632	-0.36504	0.19719
4-12 wks resolved	>=12 not resolved	0.17309	0.046258	0.001711	0.046913	0.29928
4-12 wks resolved	>=12 resolved	-0.019877	0.05596	0.9966	-0.17252	0.13277
4-12 wks resolved	Asymp-4wks resolved	-0.025385	0.032309	0.93485	-0.11352	0.062747

>=12 not resolved	no COVID	-0.25702	0.10443	0.09959	-0.54188	0.027837
>=12 not resolved	4-12 wks resolved	-0.17309	0.046258	0.001711	-0.29928	-0.046913
>=12 not resolved	>=12 resolved	-0.19297	0.057927	0.0077083	-0.35098	-0.034959
>=12 not resolved	Asymp-4wks resolved	-0.19848	0.03641	5.066e-07	-0.2978	-0.09916
>=12 resolved	no COVID	-0.06405	0.10919	0.97711	-0.3619	0.2338
>=12 resolved	4-12 wks resolved	0.019877	0.05596	0.9966	-0.13277	0.17252
>=12 resolved	>=12 not resolved	0.19297	0.057927	0.0077083	0.034959	0.35098
>=12 resolved	Asymp-4wks resolved	-0.005508	0.048352	0.99996	-0.1374	0.12639
Asymp-4wks resolved	no COVID	-0.058542	0.097678	0.97522	-0.32499	0.2079
Asymp-4wks resolved	4-12 wks resolved	0.025385	0.032309	0.93485	-0.062747	0.11352
Asymp-4wks resolved	>=12 not resolved	0.19848	0.03641	5.066e-07	0.09916	0.2978
Asymp-4wks resolved	>=12 resolved	0.005508	0.048352	0.99996	-0.12639	0.1374

Supplementary Table 7: Tiredness – Repeated Measures (Main Effects)

Source	SumSq	DF	MeanSq	F	pValue	pValueGG	pValueHF	pValueLB
(Intercept)	100.52	1	100.52	31.764	1.76E-08	1.76E-08	1.76E-08	1.76E-08
**Hospitalisation**	**37.378**	**2**	**18.689**	**5.9055**	**0.0027284**	**0.0027284**	**0.0027284**	**0.0027284**
**Intensive Care**	**23.841**	**2**	**11.921**	**3.7668**	**0.02314**	**0.02314**	**0.02314**	**0.02314**
AandE	10.365	2	5.1827	1.6376	0.19446	0.19446	0.19446	0.19446
Variant	8.8484	4	2.2121	0.69899	0.59254	0.59254	0.59254	0.59254
**Covid Duration**	**137.44**	**4**	**34.359**	**10.857**	**8.58E-09**	**8.58E-09**	**8.58E-09**	**8.58E-09**
Error	76206	24080	3.1647					

Supplementary Table 8: Tiredness – Repeated Measures (Block Effects)

Source	SumSq	DF	MeanSq	F	pValue	pValueGG	pValueHF	pValueLB
**Block**	**18.765**	**3**	**6.2552**	**23.958**	**1.72E-15**	**5.77E-13**	**5.76E-13**	**9.91E-07**
Hospitalisation:Block	1.8236	6	0.30393	1.1641	0.3223	0.32428	0.32428	0.31223
Intensive Care:Block	1.9021	6	0.31702	1.2142	0.29529	0.29995	0.29995	0.29697
AandE:Block	1.0249	6	0.17081	0.65423	0.68678	0.65327	0.65328	0.51985
Variant:Block	3.0235	12	0.25196	0.96502	0.47996	0.47023	0.47024	0.42529
COVID Duration:Block	4.7801	12	0.39834	1.5257	0.10668	0.12585	0.12584	0.19165
Error(Block)	18861	72240	0.26109					

Supplementary Table 9: Tiredness – COVID Pairwise Comparisons

Group 1	Group 2	Difference	StdErr	pValue	Lower 95% CI	Upper 95% CI
no COVID	4-12 wks resolved	-0.11573	0.11369	0.84721	-0.42584	0.19438
no COVID	>=12 not resolved	-0.24794	0.1152	0.19816	-0.56218	0.066299
no COVID	>=12 resolved	-0.1192	0.12045	0.86014	-0.44777	0.20937
no COVID	Asymp-4wks resolved	-0.0084533	0.10775	0.99999	-0.30238	0.28547

4-12 wks resolved	no COVID	0.11573	0.11369	0.84721	-0.19438	0.42584
4-12 wks resolved	>=12 not resolved	-0.13221	0.051029	0.072011	-0.27141	0.0069879
4-12 wks resolved	>=12 resolved	-0.003471	0.061732	1	-0.17186	0.16492
4-12 wks resolved	Asymp-4wks resolved	-0.10728	0.035641	0.021979	-0.2045	-0.010056
>=12 not resolved	no COVID	0.24794	0.1152	0.19816	-0.066299	0.56218
>=12 not resolved	4-12 wks resolved	0.13221	0.051029	0.072011	-0.0069879	0.27141
>=12 not resolved	>=12 resolved	0.12874	0.063902	0.25883	-0.045572	0.30305
>=12 not resolved	Asymp-4wks resolved	0.23949	0.040166	3.44E-08	0.12992	0.34905
>=12 resolved	no COVID	0.1192	0.12045	0.86014	-0.20937	0.44777
>=12 resolved	4-12 wks resolved	0.003471	0.061732	1	-0.16492	0.17186
>=12 resolved	>=12 not resolved	-0.12874	0.063902	0.25883	-0.30305	0.045572
>=12 resolved	Asymp-4wks resolved	0.11075	0.05334	0.23034	-0.03475	0.25625
Asymp-4wks resolved	no COVID	0.0084533	0.10775	0.99999	-0.28547	0.30238
Asymp-4wks resolved	4-12 wks resolved	0.10728	0.035641	0.021979	0.010056	0.2045
Asymp-4wks resolved	>=12 not resolved	-0.23949	0.040166	3.44E-08	-0.34905	-0.12992
Asymp-4wks resolved	>=12 resolved	-0.11075	0.05334	0.23034	-0.25625	0.03475

Supplementary Table 10: Motivation – Repeated Measures (Main Effects)

**Source**	**SumSq**	**DF**	**MeanSq**	**F**	**pValue**	**pValueGG**	**pValueHF**	**pValueLB**
(Intercept)	28.196	1	28.196	9.1736	0.0024579	0.0024579	0.0024579	0.0024579
Hospitalisation	6.8695	2	3.4348	1.1175	0.32711	0.32711	0.32711	0.32711
Intensive Care	7.1189	2	3.5595	1.1581	0.31411	0.31411	0.31411	0.31411
AandE	7.1073	2	3.5536	1.1562	0.3147	0.3147	0.3147	0.3147
Variant	17.672	4	4.4181	1.4374	0.21867	0.21867	0.21867	0.21867
COVID Duration	23.208	4	5.8019	1.8877	0.10954	0.10954	0.10954	0.10954
Error	74012	24080	3.0736					

Supplementary Table 11: Motivation – Repeated Measures (Block Effects)

**Source**	**SumSq**	**DF**	**MeanSq**	**F**	**pValue**	**pValueGG**	**pValueHF**	**pValueLB**
**Block**	**10.755**	**3**	**3.5851**	**11.985**	**7.68E-08**	**6.58E-07**	**6.57E-07**	**0.00053719**
Hospitalisation:Block	1.4223	6	0.23706	0.7925	0.5756	0.55531	0.55533	0.45273
Intensive Care:Block	1.5922	6	0.26536	0.88712	0.50315	0.4889	0.4889	0.41186
AandE:Block	0.88227	6	0.14704	0.49158	0.81516	0.78358	0.78361	0.61167
Variant:Block	1.4417	12	0.12014	0.40164	0.96373	0.94704	0.94705	0.80761
COVID Duration:Block	1.1699	12	0.097491	0.32592	0.98499	0.97493	0.97494	0.86075
Error(Block)	21609	72240	0.29913					

Supplementary Table 12: Motivation – COVID Pairwise Comparisons

Group 1	Group 2	Difference	StdErr	pValue	Lower 95% CI	Upper 95% CI
no COVID	4-12 wks resolved	0.045297	0.11204	0.99439	-0.26032	0.35091
no COVID	>=12 not resolved	0.094974	0.11353	0.91928	-0.21471	0.40466
no COVID	>=12 resolved	-0.0078237	0.11871	1	-0.33163	0.31598
no COVID	Asymp-4wks resolved	-0.0028335	0.10619	1	-0.2925	0.28683
4-12 wks resolved	no COVID	-0.045297	0.11204	0.99439	-0.35091	0.26032
4-12 wks resolved	>=12 not resolved	0.049677	0.05029	0.86094	-0.087501	0.18686
4-12 wks resolved	>=12 resolved	-0.053121	0.060837	0.90681	-0.21907	0.11283
4-12 wks resolved	Asymp-4wks resolved	-0.048131	0.035125	0.6468	-0.14394	0.047681
>=12 not resolved	no COVID	-0.094974	0.11353	0.91928	-0.40466	0.21471
>=12 not resolved	4-12 wks resolved	-0.049677	0.05029	0.86094	-0.18686	0.087501
>=12 not resolved	>=12 resolved	-0.1028	0.062975	0.47654	-0.27458	0.068984
>=12 not resolved	Asymp-4wks resolved	0.097808	0.039583	0.097269	-0.020578	0.20578
>=12 resolved	no COVID	0.0078237	0.11871	1	-0.31598	0.33163
>=12 resolved	4-12 wks resolved	0.053121	0.060837	0.90681	-0.11283	0.21907
>=12 resolved	>=12 not resolved	0.1028	0.062975	0.47654	-0.068984	0.27458
>=12 resolved	Asymp-4wks resolved	0.0049901	0.052566	0.99998	-0.1384	0.14838
Asymp-4wks resolved	no COVID	0.0028335	0.10619	1	-0.28683	0.2925
Asymp-4wks resolved	4-12 wks resolved	0.048131	0.035125	0.6468	-0.047681	0.14394
Asymp-4wks resolved	>=12 not resolved	-0.097808	0.039583	0.097269	-0.20578	0.010167
Asymp-4wks resolved	>=12 resolved	-0.0049901	0.052566	0.99998	-0.14838	0.1384

Supplementary Table 13: Reaction Time – Repeated Measures (Main Effects)

Source	SumSq	DF	MeanSq	F	pValue	pValueGG	pValueHF	pValueLB
(Intercept)	4.7388	1	4.7388	1.7269	0.18882	0.18882	0.18882	0.18882
Hospitalisation	1.1349	2	0.56743	0.20678	0.8132	0.8132	0.8132	0.8132
Intensive Care	2.1711	2	1.0855	0.39559	0.67328	0.67328	0.67328	0.67328
AandE	0.6684	2	0.3342	0.12179	0.88533	0.88533	0.88533	0.88533
Variant	3.1689	4	0.79223	0.28871	0.88547	0.88547	0.88547	0.88547
**COVID Duration**	**27.687**	**4**	**6.9216**	**2.5224**	**0.038971**	**0.038971**	**0.038971**	**0.038971**
Error	66077	24080	2.744					

Supplementary Table 14: Reaction Time – Repeated Measures (Block Effects)

Source	SumSq	DF	MeanSq	F	pValue	pValueGG	pValueHF	pValueLB
Block	2.9977	3	0.99922	2.3952	0.06622	0.067787	0.067778	0.12172
Hospitalisation:Block	2.5758	6	0.4293	1.0291	0.40395	0.40325	0.40325	0.35735
Intensive Care:Block	2.0205	6	0.33674	0.8072	0.56408	0.56139	0.5614	0.44612
AandE:Block	2.736	6	0.45599	1.093	0.36364	0.36346	0.36346	0.33521

Variant:Block	4.4796	12	0.3733	0.89483	0.5515	0.54953	0.54954	0.46594
COVID Duration:Block	2.7073	12	0.22561	0.54079	0.88942	0.88589	0.88591	0.70578
Error(Block)	30137	72240	0.41717					

Supplementary Table 15: Reaction Time – COVID Pairwise Comparisons

Group 1	Group 2	Difference	StdErr	pValue	Lower 95% CI	Upper 95% CI
no COVID	4-12 wks resolved	-0.026903	0.10586	0.99908	-0.31567	0.26187
no COVID	>=12 not resolved	-0.15823	0.10727	0.5788	-0.45084	0.13438
no COVID	>=12 resolved	-0.096284	0.11216	0.91196	-0.40224	0.20967
no COVID	Asymp-4wks resolved	-0.050477	0.10034	0.98709	-0.32417	0.22322
4-12 wks resolved	no COVID	0.026903	0.10586	0.99908	-0.26187	0.31567
4-12 wks resolved	>=12 not resolved	-0.13132	0.047517	0.045267	-0.26094	-0.0017073
4-12 wks resolved	>=12 resolved	-0.069381	0.057483	0.74736	-0.22618	0.087419
4-12 wks resolved	Asymp-4wks resolved	-0.023575	0.033188	0.95422	-0.1141	0.066955
>=12 not resolved	no COVID	0.15823	0.10727	0.5788	-0.13438	0.45084
>=12 not resolved	4-12 wks resolved	0.13132	0.047517	0.045267	0.0017073	0.26094
>=12 not resolved	>=12 resolved	0.061942	0.059503	0.8363	-0.10037	0.22425
>=12 not resolved	Asymp-4wks resolved	0.10775	0.037401	0.032382	0.0057265	0.20977
>=12 resolved	no COVID	0.096284	0.11216	0.91196	-0.20967	0.40224
>=12 resolved	4-12 wks resolved	0.069381	0.057483	0.74736	-0.087419	0.22618
>=12 resolved	>=12 not resolved	-0.061942	0.059503	0.8363	-0.22425	0.10037
>=12 resolved	Asymp-4wks resolved	0.045807	0.049668	0.88842	-0.089677	0.18129
Asymp-4wks resolved	no COVID	0.050477	0.10034	0.98709	-0.22322	0.32417
Asymp-4wks resolved	4-12 wks resolved	0.023575	0.033188	0.95422	-0.066955	0.1141
Asymp-4wks resolved	>=12 not resolved	-0.10775	0.037401	0.032382	-0.20977	-0.0057265
Asymp-4wks resolved	>=12 resolved	-0.045807	0.049668	0.88842	-0.18129	0.089677

Linear Regression

Supplementary Table 16: DVT Accuracy – ANOVA summary

Source	SumSq	DF	MeanSq	F	p-value
Hospital_1	0.23439	2	0.11719	0.15429	0.85702
Intensive_care_1	2.4553	2	1.2276	1.6163	0.19866
AandE_1	0.2819	2	0.14095	0.18557	0.83063
CAT_12	18.814	4	4.7036	6.1926	5.6255e-05
Tiredness	15.166	1	15.166	19.967	7.9153e-06
Motivation	728.85	1	728.85	959.58	1.2388e-206
RT	4476.1	1	4476.1	5893.1	<0.001

Error (residual)	18291	24081	0.75955

Supplementary Table 17: DVT Accuracy – Regression Estimates

Predictor	Estimate	SE	t-stat	p-value	CI lower	CI upper
(Intercept)	0.019384	0.019248	1.0071	0.3139	-0.018342	0.057111
Hospital_1=Yes	-0.02160	0.074373	-0.29043	0.77149	-0.16738	0.12418
Hospital_1=unknown	-0.090593	0.19126	-0.47365	0.63575	-0.46548	0.28430
Intensive_care_1=Yes	-0.21423	0.13409	-1.5977	0.11012	-0.47705	0.048587
Intensive_care_1=unknown	0.17182	0.20829	0.8249	0.40944	-0.23644	0.58008
AandE_1=Yes	0.014928	0.075187	0.19855	0.84262	-0.13244	0.16230
AandE_1=unknown	-0.079182	0.13751	-0.57582	0.56474	-0.34871	0.19035
CAT_12 (4–12 wks)	-0.061356	0.038673	-1.5866	0.11263	-0.13716	0.014445
CAT_12 (>=12 not resolved)	-0.20731	0.042394	-4.8901	1.0145e-06	-0.29040	-0.12421
CAT_12 (>=12 resolved)	-0.030722	0.053811	-0.57093	0.56805	-0.13619	0.07475
CAT_12 (Asymptomatic-4wks)	-0.02978	0.02007	-1.4838	0.13788	-0.069119	0.0095593
Tiredness	-0.027114	0.006068	-4.4684	7.9153e-06	-0.039008	-0.015221
Motivation	0.18783	0.006034	30.977	1.2388e-206	0.17594	0.19971
RT	-0.43244	0.005633	-76.767	<0.001	-0.44348	-0.42140

Supplementary Table 18: Reaction Time – ANOVA Summary

Source	SumSq	DF	MeanSq	F	pValue
Hospital_1	0.13049	2	0.065244	0.081647	0.9216
Intensive_care_1	0.03982	2	0.01991	0.024915	0.97539
AandE_1	0.040193	2	0.020097	0.025149	0.97516
CAT_12	5.119	4	1.2797	1.6015	0.17086
Tiredness	1.4666	1	1.466	1.8345	0.17561
Motivation	28.687	1	28.687	35.899	2.1073e-09
Attention	4709.2	1	4709.2	5893.1	0
Error	19243	24081	0.79911		

Supplementary Table 19: Reaction Time – Regression Estimates

Predictor	Estimate	SE	tStat	pValue	CI_Lower	CI_Upper
(Intercept)	0.023708	0.019742	1.2009	0.22981	-0.014988	0.062404
Hospital_1_Yes	0.030085	0.076285	0.39438	0.69331	-0.11944	0.17961
Hospital_1_unknown	-0.017251	0.19618	-0.087932	0.92993	-0.40178	0.36728
Intensive_care_1_Yes	-0.028292	0.13754	-0.2057	0.83702	-0.29788	0.24129
Intensive_care_1_unknown	-0.01851	0.21365	-0.086639	0.93096	-0.43727	0.40025
AandE_1_Yes	0.00013416	0.07712	0.0017396	0.99861	-0.15103	0.15129
AandE_1_unknown	0.031632	0.14105	0.22427	0.82255	-0.24483	0.30809
CAT_12_4-12wks	-0.082112	0.039665	-2.0701	0.038451	-0.15986	-0.004366
CAT_12_>=12_not_resolved	-0.048916	0.043504	-1.1244	0.26085	-0.13419	0.036354
CAT_12_>=12_resolved	-0.0062955	0.055194	-0.11406	0.90919	-0.11448	0.10189
CAT_12_Asymp-4wks	-0.042765	0.020586	-2.0774	0.037773	-0.083114	-0.0024159
Tiredness	0.0084331	0.0062263	1.3544	0.17561	-0.0037708	0.020637
Motivation	0.03797	0.0063372	5.9916	2.1073e-09	0.025549	0.050392
Attention	-0.45496	0.0059266	-76.767	0	-0.46658	-0.44335

Supplementary Table 20: Immediate Memory – ANOVA Summary

Source	SumSq	DF	MeanSq	F	pValue
Hospital_1	6.7236	2	3.3618	3.4158	0.032865
Intensive_care_1	0.56038	2	0.28019	0.28469	0.75225
AandE_1	0.58779	2	0.2939	0.29862	0.74185
variant_1	11.897	4	2.9742	3.022	0.016726
CAT_12	17.692	3	5.8973	5.9921	0.00044619
Tiredness	26.648	1	26.648	27.076	1.9724e-07
Attention	244.93	1	244.93	248.86	8.741e-56

Motivation	0.022376	1	0.022376	0.022376	0.88015
Error	23696	24077	0.98419		

Supplementary Table 21: Immediate Memory – Regression Estimates

Predictor	Estimate	SE	tStat	pValue	CI_Lower	CI_Upper
(Intercept)	0.020738	0.021929	0.94566	0.34433	-0.022245	0.063721
Hospital_1_Yes	-0.20882	0.084881	-2.4602	0.013893	-0.3752	-0.042453
Hospital_1_unknown	0.19185	0.21772	0.88119	0.37822	-0.23489	0.61859
Intensive_care_1_Yes	-0.10135	0.15282	-0.66319	0.50722	-0.40089	0.19819
Intensive_care_1_unknown	-0.085293	0.2371	-0.35973	0.71905	-0.55003	0.37944
AandE_1_Yes	-0.026634	0.085713	-0.31073	0.75601	-0.19464	0.14137
AandE_1_unknown	-0.1108	0.15653	-0.70787	0.47903	-0.41762	0.19601
variant_1_Alpha	0.21314	0.12277	1.7361	0.08256	-0.027496	0.45377
variant_1_Delta	0.22142	0.12005	1.8443	0.065147	-0.013894	0.45672
variant_1_Omicron	0.27449	0.11924	2.302	0.021345	0.040771	0.50821
variant_1_Wild	0.23926	0.12188	1.9631	0.049651	0.00036455	0.47816
CAT_12_4-12wks	-0.26826	0.1268	-2.1155	0.034396	-0.5168	-0.019713
CAT_12_>=12_not_resolved	-0.45166	0.12851	-3.5144	0.00044149	-0.70355	-0.19976
CAT_12_>=12_resolved	-0.37625	0.13435	-2.8005	0.005107	-0.63958	-0.11291
CAT_12_Asymp-4wks	-0.27404	0.12018	-2.2803	0.022601	-0.5096	-0.038481
Tiredness	-0.035957	0.0069102	-5.2034	1.9724e-07	-0.049502	-0.022413
Attention	0.10377	0.006578	15.775	8.741e-56	0.090878	0.11666
Motivation	-0.0010605	0.0070336	-0.15078	0.88015	-0.014847	0.012726

Supplementary Table 22: Delayed Memory – ANOVA Summary

Source	SumSq	DF	MeanSq	F	pValue
Hospital_1	9.0831	2	4.5415	4.6292	0.009771

Intensive_care_1	1.4595	2	0.72973	0.74382	0.47531
AandE_1	0.66467	2	0.33234	0.33875	0.71266
variant_1	13.843	4	3.4609	3.5277	0.0069606
CAT_12	15.628	3	5.2094	5.31	0.0011748
Tiredness	34.327	1	34.327	34.989	3.3597e-09
Attention	281	1	281	286.42	7.0093e-64
Motivation	0.32208	1	0.32208	0.3283	0.56667
Error	23621	24077	0.98106		

Supplementary Table 23: Delayed Memory – Regression Estimates

Predictor	Estimate	SE	tStat	pValue	CI_Lower	CI_Upper
(Intercept)	0.019936	0.021894	0.91057	0.36253	-0.022978	0.062851
Hospital_1_Yes	-0.23525	0.084746	-2.776	0.0055075	-0.40136	-0.069147
Hospital_1_unknown	0.27047	0.21737	1.2443	0.21341	-0.15559	0.69653
Intensive_care_1_Yes	-0.1615	0.15288	-1.0585	0.28984	-0.46057	0.13756
Intensive_care_1_unknown	-0.14337	0.23672	-0.60562	0.54477	-0.60736	0.32063
AandE_1_Yes	-0.007259	0.085576	-0.084825	0.9324	-0.17499	0.16048
AandE_1_unknown	-0.12797	0.15628	-0.8188	0.41291	-0.43429	0.17836
variant_1_Alpha	0.13432	0.12257	1.0958	0.27317	-0.10593	0.37457
variant_1_Delta	0.14236	0.11986	1.1877	0.23496	-0.092576	0.37729
variant_1_Omicron	0.2061	0.11905	1.7312	0.083427	-0.027245	0.43945
variant_1_Wild	0.14371	0.12169	1.181	0.23763	-0.094809	0.38223
CAT_12_4-12wks	-0.24161	0.1266	-1.9085	0.056344	-0.48976	0.0065337
CAT_12_>=12_not_resolved	-0.36383	0.12831	-2.8355	0.0045788	-0.61532	-0.11233
CAT_12_>=12_resolved	-0.2988	0.13414	-2.2275	0.02592	-0.56171	-0.035879
CAT_12_Asymp-4wks	-0.19836	0.11999	-1.6531	0.09832	-0.43354	0.03683

Tiredness	-0.04081	0.0068993	-5.9152	3.3597e-09	-0.054333	-0.027287
Attention	0.11115	0.0065675	16.924	7.0093e-64	0.098276	0.12402
Motivation	0.0040236	0.0070224	0.57297	0.56667	-0.0097407	0.017788

Stepwise Regression

Supplementary Table 24: Stepwise Modelling – Attention Accuracy (Coefficients)

Term	Estimate	SE	tStat	pValue	CI_Lower	CI_Upper
(Intercept)	-0.39041	0.016088	-24.267	1.5E-128	-0.42194	-0.35888
Motivation	0.008718	0.000306	28.505	8.4E-176	0.008119	0.009318
RT	-0.31146	0.013198	-23.6	9.4E-122	-0.33733	-0.2856
BrainFog	-0.07587	0.016911	-4.4864	7.28E-06	-0.10902	-0.04272
PoorMemory	-0.21955	0.039106	-5.6141	2E-08	-0.2962	-0.1429
Motivation:RT	-0.0027	0.000264	-10.232	1.6E-24	-0.00322	-0.00218
Motivation:PoorMemory	0.00327	0.000773	4.2286	2.36E-05	0.001754	0.004785

Supplementary Table 25: Stepwise Modelling – Attention Accuracy (Model Fit)

N	DF_Error	RMSE	R_squared	Adj_R_squared	F_statistic	F_pValue
24095	24088	0.87	0.243	0.243	1290	0

Supplementary Table 26: Stepwise Modelling – Immediate Memory (Coefficients)

Term	Estimate	SE	tStat	pValue	CI_Lower	CI_Upper
(Intercept)	0.10398	0.017838	5.8293	5.64E-09	0.069018	0.13894
Hospitalisation	-0.28323	0.078358	-3.6145	0.000302	-0.43681	-0.12964
Tiredness	-0.00172	0.000317	-5.4163	6.14E-08	-0.00234	-0.0011
Attention	0.091661	0.007017	13.063	7.28E-39	0.077907	0.10541
PoorMemory	0.060101	0.018382	-3.2696	0.001079	-0.09613	-0.02407
Attention:PoorMemory	0.07312	0.017577	4.1599	3.2E-05	0.038667	0.10757

Supplementary Table 27: Stepwise Modelling – Immediate Memory (Model Fit)

N	DF_Error	RMSE	R_squared	Adj_R_squared	F_statistic	F_pValue
24095	24089	0.992	0.0156	0.0154	76.2	1.54E-79

Supplementary Table 28: Stepwise Modelling – Delayed Memory (Coefficients)

Term	Estimate	SE	tStat	pValue	CI_Lower	CI_Upper
(Intercept)	0.12217	0.017812	6.8591	7.1E-12	0.087262	0.15709
Hospitalisation	-0.32446	0.078245	-4.1467	3.38E-05	-0.47782	-0.17109
Tiredness	-0.00203	0.000317	-6.3987	1.6E-10	-0.00265	-0.00141
Attention	0.10246	0.007007	14.623	3.22E-48	0.088727	0.11619
PoorMemory	-0.07327	0.018355	-3.9918	6.58E-05	-0.10925	-0.03729
Attention:PoorMemory	0.056833	0.017552	3.238	0.001206	0.02243	0.091237

Supplementary Table 29: Stepwise Modelling – Delayed Memory (Model Fit)

N	DF_Error	RMSE	R_squared	Adj_R_squared	F_statistic	F_pValue
24095	24089	0.991	0.0184	0.0182	90.4	1.55E-94

Path Analysis

```
model3 <- '

Tiredness ~ f1*COVID_duration + f2*Hospitalised + f3*BrainFog + f4*PoorMemory
Motivation ~ m1*Tiredness + c1*COVID_duration + c2*Hospitalised + c3*BrainFog + c4*PoorMemory
Attention ~  a1*Motivation + d1*COVID_duration + d2*Hospitalised + d3*BrainFog + d4*PoorMemory
ImmediateMemory ~ i1*Attention + e1*COVID_duration + e2*Hospitalised + e3*BrainFog + e4*PoorMemory
DelayedMemory ~ g1*ImmediateMemory + h1*COVID_duration + h2*Hospitalised + h3*BrainFog + h4*PoorMemory

ind_COVID_tired := f1*m1*a1*i1*g1
ind_Hosp_tired := f2*m1*a1*i1*g1
ind_BF_tired := f3*m1*a1*i1*g1
ind_PM_tired := f4*m1*a1*i1*g1

ind_COVID_mot := c1*m1*a1*i1*g1
ind_Hosp_mot := c2*m1*a1*i1*g1
ind_BF_mot := c3*m1*a1*i1*g1
ind_PM_mot := c4*m1*a1*i1*g1

ind_COVID_att := d1*m1*a1*i1*g1
ind_Hosp_att := d2*m1*a1*i1*g1
ind_BF_att := d3*m1*a1*i1*g1
ind_PM_att := d4*m1*a1*i1*g1

ind_COVID_att_mem := d1*g1
ind_Hosp_att_mem := d2*g1
ind_BF_att_mem := d3*g1
ind_PM_att_mem := d4*g1
'
```

Supplementary Figure 3: Path Model Specification: PPM1 model specification including direct and indirect paths.

Supplementary Table 30: Indirect paths for Model 3(PPM1)

Parameter	Estimate	Std.Err	z-value	p-value	CI Lower	CI Upper	Std.lv	Std.all
ind_COVID_tird	-0.000	0.000	-1.262	0.207	-0.001	0.000	-0.000	-0.000
ind_Hosp_tired	-0.002	0.001	-1.618	0.106	-0.004	0.001	-0.002	-0.001

ind_BF_tired	-0.002	0.001	-2.804	0.005	-0.004	-0.001	-0.002	-0.001
ind_PM_tired	-0.001	0.001	-1.256	0.209	-0.002	0.000	-0.001	-0.000
ind_COVID_mot	-0.000	0.000	-0.227	0.820	-0.001	0.000	-0.000	-0.000
ind_Hosp_mot	-0.000	0.001	-0.310	0.757	-0.002	0.001	-0.000	-0.000
ind_BF_mot	0.000	0.001	0.515	0.606	-0.001	0.001	0.000	0.000
ind_PM_mot	0.000	0.001	0.700	0.484	-0.001	0.001	0.000	0.000
ind_COVID_att	0.001	0.000	2.160	0.031	0.000	0.002	0.001	0.001
ind_Hosp_att	0.001	0.001	0.752	0.452	-0.001	0.003	0.001	0.000
ind_BF_att	0.000	0.000	0.700	0.484	-0.001	0.001	0.000	0.000
ind_PM_att	0.001	0.001	0.898	0.369	-0.001	0.002	0.001	0.001
ind_COVID_tt_m	-0.057	0.022	-2.608	0.009	-0.099	-0.013	-0.057	-0.052
ind_Hosp_tt_mm	-0.067	0.086	-0.779	0.436	-0.189	0.104	-0.067	-0.017
ind_BF_att_mem	-0.037	0.051	-0.732	0.464	-0.136	0.065	-0.037	-0.019
ind_PM_att_mem	-0.051	0.055	-0.935	0.350	-0.158	0.057	-0.051	-0.025

Supplementary Table 31: Covariances from Model 3 (PPM1)

Parameter	Estimate	Std.Err	z-value	p-value	CI Lower	CI Upper	Std.lv	Std.all
COVID_duration ~~ Hospitalised	0.010	0.006	1.676	0.094	-0.002	0.021	0.010	0.044
COVID_duration ~~ BrainFog	0.094	0.011	8.517	0.000	0.072	0.116	0.094	0.213
COVID_duration ~~ PoorMemory	0.077	0.011	7.237	0.000	0.056	0.098	0.077	0.184
Hospitalised ~~ BrainFog	0.007	0.003	2.374	0.018	0.002	0.014	0.007	0.063
Hospitalised ~~ PoorMemory	0.004	0.002	2.277	0.023	0.001	0.007	0.004	0.071
BrainFog ~~ PoorMemory	0.144	0.005	27.749	0.000	0.134	0.154	0.144	0.624

Supplementary Table 32: Intercepts from Model 3 (PPM1)

Parameter	Estimate	Std.Err	z-value	p-value	CI Lower	CI Upper	Std.lv	Std.all
Tiredness	-0.056	0.089	-0.634	0.526	-0.236	0.114	-0.056	-0.055
Motivation	0.030	0.081	0.367	0.714	-0.127	0.193	0.030	-0.029
Attention	0.247	0.083	2.957	0.003	0.078	0.408	0.247	0.249
ImmediateMemory	0.198	0.053	3.731	0.000	0.094	0.302	0.180	0.177
DelayedMemory	-0.066	0.053	-1.251	0.211	-0.168	0.038	-0.066	-0.066
COVID_duration	0.375	0.029	12.497	0.000	0.319	0.432	0.375	0.363
Hospitalised	0.061	0.006	9.858	0.000	0.050	0.074	0.062	0.256
BrainFog	0.416	0.006	69.035	0.000	0.392	0.441	0.416	0.844
PoorMemory	0.323	0.012	27.172	0.000	0.300	0.346	0.323	0.691

Supplementary Table 33: Variances from Model 3(PPM1)

Parameter	Estimate	Std.Err	z-value	p-value	CI Lower	CI Upper	Std.lv	Std.all
Tiredness	1.006	0.034	30.035	0.000	0.939	1.070	1.006	0.964
Motivation	0.877	0.038	23.242	0.000	0.803	0.949	0.877	0.847
Attention	0.924	0.035	26.168	0.000	0.854	0.992	0.924	0.937
ImmediateMemory	0.995	0.038	26.424	0.000	0.916	1.064	0.995	0.967
DelayedMemory	0.356	0.032	11.253	0.000	0.317	0.395	0.356	0.363
COVID_duration	0.795	0.011	70.918	0.000	0.773	0.815	0.795	1.000
Hospitalised	0.058	0.002	24.000	0.000	0.048	0.069	0.058	1.000
BrainFog	0.243	0.002	114.668	0.000	0.238	0.246	0.243	1.000
PoorMemory	0.219	0.004	51.979	0.000	0.210	0.226	0.219	1.000

Supplementary Table 34: R-squared values from Model 3(PPM1)

Variable	Estimate
**Tiredness**	0.036
**Motivation**	0.153
**Attention**	0.163
**ImmediateMemory**	0.033
**DelayedMemory**	0.637

Supplementary Table 35: Regressions on Tiredness

Predictor	Estimate	Std.Err	z-value	p-value	CI Lower	CI Upper	Std.lv	Std.all
COVID_Duration	0.041	0.030	1.371	0.170	-0.017	0.101	0.041	0.035
Hospitalised (f2)	0.210	0.115	1.826	0.068	-0.012	0.436	0.210	0.049
BrainFog (f3)	0.287	0.065	4.403	0.000	0.162	0.417	0.287	0.138
PoorMemory (f4)	0.096	0.070	1.369	0.171	-0.040	0.233	0.096	0.044

Supplementary Table 36: Regressions on Motivation

Predictor	Estimate	Std.Err	z-value	p-value	CI Lower	CI Upper	Std.lv	Std.all
Tiredness (m1)	-0.382	0.030	-12.632	0.000	-0.441	-0.324	-0.382	-0.384
COVID_Duration	0.006	0.027	0.234	0.815	-0.048	0.060	0.006	0.006
Hospitalised (c2)	0.034	0.107	0.321	0.748	-0.167	0.247	0.034	0.008
BrainFog (c3)	-0.030	0.065	-0.528	0.598	-0.156	0.092	-0.030	-0.017
PoorMemory (c4)	-0.050	0.068	-0.731	0.465	-0.181	0.085	-0.050	-0.023

Supplementary Table 37: Regressions on Attention

Predictor	Estimate	Std.Err	z-value	p-value	CI Lower	CI Upper	Std.lv	Std.all
Motivation (a1)	0.221	0.025	8.717	0.000	0.171	0.270	0.221	0.226
COVID_Duration	-0.073	0.028	-2.613	0.009	-0.127	-0.017	-0.073	-0.066
Hospitalised (d2)	-0.087	0.111	-0.778	0.436	-0.309	0.133	-0.087	-0.021
BrainFog (d3)	-0.048	0.066	-0.732	0.464	-0.175	0.082	-0.048	-0.024
PoorMemory (d4)	-0.066	0.070	-0.934	0.350	-0.204	0.073	-0.066	-0.031

Supplementary Table 38: Regressions on Immediate Memory

Predictor	Estimate	Std.Err	z-value	p-value	CI Lower	CI Upper	Std.lv	Std.all
Attention (i1)	0.117	0.027	4.362	0.000	0.064	0.169	0.117	0.114
COVID_Duration	-0.082	0.029	-2.860	0.004	-0.139	-0.025	-0.082	-0.072
Hospitalised (e2)	0.270	0.105	2.583	0.010	0.071	0.471	0.270	0.064
BrainFog (e3)	-0.035	0.071	-0.489	0.625	-0.173	0.103	-0.035	-0.017
PoorMemory (e4)	-0.119	0.074	-1.594	0.111	-0.266	0.030	-0.119	-0.055

Supplementary Table 39: Regressions on Delayed Memory

Predictor	Estimate	Std.Err	z-value	p-value	CI Lower	CI Upper	Std.lv	Std.all
ImmediateMemory	0.775	0.015	51.350	0.000	0.744	0.805	0.775	0.794
COVID_Duration	0.008	0.017	0.475	0.635	-0.026	0.042	0.008	0.007
Hospitalised (h2)	-0.115	0.072	-1.600	0.110	-0.258	0.025	-0.115	-0.028
BrainFog (h3)	-0.012	0.045	-0.275	0.783	-0.101	0.077	-0.012	-0.006
PoorMemory (h4)	-0.033	0.045	-0.728	0.466	-0.123	0.053	-0.033	-0.016
